# Integrative single-cell analysis of transcriptome, DNA methylome and chromatin accessibility in mouse oocytes

**DOI:** 10.1038/s41422-018-0125-4

**Published:** 2018-12-18

**Authors:** Chan Gu, Shanling Liu, Qihong Wu, Lin Zhang, Fan Guo

**Affiliations:** 1 Center for Translational Medicine, Ministry of Education Key Laboratory of Birth Defects and Related Diseases of Women and Children, Department of Obstetrics and Gynecology, West China Second University Hospital, Sichuan University, Chengdu, Sichuan 610041 China; 2 Ministry of Education Key Laboratory of Bio-resource and Eco-environment, College of Life Sciences, Sichuan University, Chengdu, Sichuan 610041 China

**Keywords:** Chromatin remodelling, Chromatin analysis, Reprogramming

## Abstract

Oocyte growth is a key step in forming mature eggs that are ready to be fertilized. The states and modifications of chromatin represent critical sources of information for this process. However, the dynamics and interrelations of these chromatin characteristics remain elusive. In this study, we developed an improved scCOOL-seq technique (iscCOOL-seq), which is a multi-omics, single-cell and single-base resolution method with high mapping rates, and explored the chromatin accessibility landscape and its relationship to DNA methylation in growing mouse oocytes. The most dramatic change in chromatin accessibility occurs during oocyte growth initiation, accompanied with prominent transcriptome alterations and an elevated variation in DNA methylation levels among individual oocytes. Unlike CpG islands (CGIs), partially methylated domains (PMDs) are associated with a low density of nucleosome-depleted regions (NDRs) during the whole maturation period. Surprisingly, highly expressed genes are usually associated with NDRs at their transcriptional end sites (TESs). In addition, genes with de novo methylated gene bodies during oocyte maturation are already open at their promoters before oocyte growth initiation. Furthermore, epigenetic and transcription factors that might be involved in oocyte maturation are identified. Our work paves the way for dissecting the complex, yet highly coordinated, epigenetic alterations during mouse oocyte growth and the establishment of totipotency.

## Introduction

The development of the female germline undergoes several key steps to generate mature oocytes that are ready for fertilization; these steps include the formation of primordial germ cells (PGCs) from a limited number of epiblast founders, the transition from mitosis to meiosis after the completion of epigenetic reprogramming in the PGCs, and oogenesis during postnatal growth^1,2^. Many studies have used mouse models to dissect the fundamental signalling pathways and important epigenetic features involved in PGC formation, migration and colonization^3–7^. Recently, much effort has been made to extend our understanding of germline development to human PGCs^8–18^. However, the epigenetic features of oocyte growth (oogenesis), especially the chromatin configuration and its relationship with essential chromatin modifications, such as DNA methylation, remain elusive.

In the female germline, de novo DNA methylation takes place in growing oocytes during postnatal growth^19^. Targeted sequences will be methylated during this de novo DNA methylation process, including the germline differentially methylated regions of imprinted genes, thus forming oocyte-specific DNA methylation profiles^19^. Past studies have revealed that DNA methyltransferase, Dnmt3a and its cofactor Dnmt3l, are involved in de novo DNA methylation of oocytes^19^. The role of chromatin status, such as histone modifications, in this process has also been studied recently^20^. However, the chromatin landscape and its dynamics during oocyte growth remain unexplored. Several studies have taken advantage of ATAC-seq or DNase-seq with hundreds of cells to capture the profile of chromatin accessibility in preimplantation embryos^21–24^. However, due to an extremely limited cell source, it is difficult to explore the chromatin accessibility landscape during oogenesis.

Advances in single-cell epigenome sequencing technologies, which can detect chromatin modifications or structures at single-cell resolution, have greatly facilitated our exploration of the epigenetic heterogeneity of cell populations^25–28^. Recently, we and others have developed multiple single-cell multi-omics sequencing technologies, termed scCOOL-seq^29,30^, scNOMe-seq^31^ and scNMT-seq^32^, that can simultaneously analyse DNA methylation and chromatin accessibility. Besides, these methods can detect both open and closed regions throughout the genome. However, all these methods adopt the traditional post-bisulfite adaptor tagging (PBAT) strategy to capture single-cell DNA methylome. This leads to low mapping rates (22.01% on average), which increase the sequencing costs and limit the application of these methods.

In this study, we improve the scCOOL-seq technique by developing a tailing- and ligation-free method for single cells (TAILS), which simplifies the steps in constructing single-cell DNA methylome libraries and elevates the mapping efficiencies to 62.26% on average in each individual cells. Using the improved single-cell COOL-seq technique (iscCOOL-seq), which is a multi-omics, single-cell and single-base resolution method with high mapping rates, we explored the chromatin accessibility landscape and its relationship to DNA methylation in the non-growing oocyte (NGO), growing oocyte (GO1-3), and fully-grown oocyte (FGO) stages. In total, we sequenced 774 iscCOOL-seq libraries, among which 40 are generated from single mouse ES cells, 20 are from single MII oocytes and the rest are from oocytes in five different growth stages (*n* = 160 for NGO, *n* = 148 for GO1, *n* = 139 for GO2, *n* = 125 for GO3 and *n* = 142 for FGO). Additionally, single-cell RNA-seq of 296 single oocytes (see the Materials and Methods) was performed to associate the chromatin accessibility status with gene expression level during mouse oocyte growth.

## Results

### Establishment of improved single-cell COOL-seq method

Firstly, we used the iscCOOL-seq technique based on the TAILS method (Supplementary information, Fig. S1a) to generate libraries from 40 single mouse ES cells and achieved a mapping rate of 74.55% on average, much higher than that of our previous scCOOL-seq (22.01% on average, Supplementary information, Fig. S1b; Table S1; *P*-value = 1.4 × 10^−27^, two-tailed Student’s *t*-test). The general patterns of chromatin accessibility around nucleosome-depleted regions (NDRs) or transcription start sites (TSSs) in mouse ES cells were very similar comparing iscCOOL-seq and scCOOL-seq data, as shown from 25,243 wider NDRs (250 bp on average, *P*-value = 8.0 × 10^−20^, two-tailed Student’s *t*-test against 38,054 narrow NDRs with an average length of 188 bp) and 10,488 accessible promoters (Supplementary information, Fig. S1c). Moreover, we used iscCOOL-seq technique to generate 20 single-cell libraries from mouse MII oocytes and compared the accuracy of DNA methylation detection with scCOOL-seq and scBS-seq at single-cell and single-base level (Supplementary information, Fig. S1d). We found that individual MII oocytes, either from iscCOOL-seq, scCOOL-seq or scBS-seq dataset, were clustered together or separated from mouse ES cells (Supplementary information, Fig. S1d), whereas individual ES cells, which were separated from MII oocytes, were clustered together and divided into two subgroups (Supplementary information, Fig. S1d). To further confirm the detection of chromatin accessibility by iscCOOL-seq, we checked 8 representative loci, *Klf5*, *Oct4*, *Tbx3*, *Nanog*, *Zfp53*, *Uhrf1* and another two, whose accessibility had been defined already in mouse ES cells by three different methods (DNase-seq, scCOOL-seq and liDNaseI-qPCR) in our recent study^29^. The iscCOOL-seq showed a robust and highly accurate detection of open or closed chromatins at these 8 representative loci (Supplementary information, Fig. S1e). Together, the improved scCOOL-seq, which was with elevated mapping efficiency, could detect both chromatin accessibility and DNA methylation with robustness and high accuracy.

### Global features of chromatin accessibility in growing mouse oocytes revealed by iscCOOL-seq

**Fig. 1:**
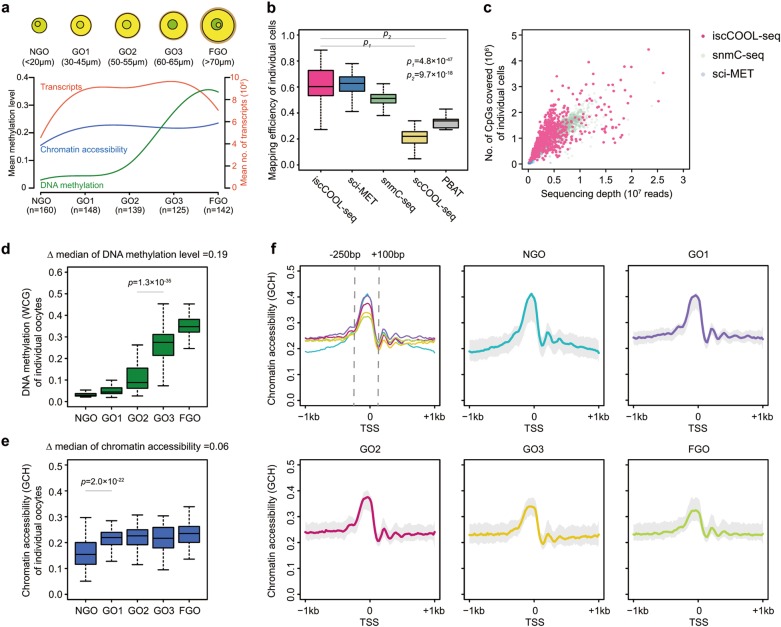
Single-cell analysis of both chromatin accessibility and DNA methylation in growing mouse oocytes. **a** A diagram of the number and diameters of growing oocytes used at each stage. The lower panel shows the mean of chromatin accessibility (GCH methylation level), DNA methylation (WCG methylation level) and the abundance of transcripts at each stage. **b** Comparison of the mapping efficiency among iscCOOL-seq, sci-MET^34^, snmC-seq^33^, scCOOL-seq via the PBAT strategy and the traditional PBAT^29^. **c** Comparison of the CpG coverage per sequencing depth among iscCOOL-seq, sci-MET and snmC-seq. **d** Boxplot of the DNA methylation levels (WCG) in individual oocytes. **e** Boxplot of the chromatin accessibility (GCH) in individual oocytes. **f** Chromatin accessibility around TSS (±1 kb) in aggregated single oocytes and individual oocytes. Shading denotes for the 25th and 75th percentile of chromatin accessibility among the single oocytes. P-values were defined by the two-tailed Student’s *t*-test

Next, we sequenced 714 growing mouse oocytes covering five stages and achieved a mapping rate of 62.26% on average (Fig. 1a, b). This alignment rate was also significantly higher than that achieved using the traditional PBAT method (*P*-value = 9.7 × 10^−18^, two-tailed Student’s *t*-test) and was comparable to those obtained by the snmC-seq^33^ and sci-MET^34^ methods, which were both recently developed for single-cell whole-genome bisulfite sequencing (Fig. 1b). Furthermore, the numbers of CG dinucleotides covered per sequencing depth in each single cell were also comparable to those covered using the snmC-seq and sci-MET methods (Fig. 1c). Sequencing the libraries with an average of 1.39 G bases per single cell (with an average of 2,621,450 unique mapped reads per single cell) covered a mean of 84.7% of the GCH fractions of the genome in aggregated single oocytes (Supplementary information, Fig. S2a) and detected DNA methylation levels that highly correlated with the results got by the traditional PBAT^35,36^ method (*R* = 0.90 in the GO3 stage and *R* = 0.93 in FGOs by the Spearman’s correlation coefficient) (Supplementary information, Fig. S2b).

To further analyse the chromatin features of single oocytes, cells with fewer than two million of covered GCH sites were excluded, leaving 667 single oocytes satisfying this criterion (93.41% efficiency). The DNA methylation level increased during oocyte maturation (Fig. 1d; Supplementary information, Fig. S2c), and the most dramatic increase occurred between the GO2 and GO3 stages (Δ median DNA methylation = 0.19, P-value = 1.3 × 10^−35^, two-tailed Student’s *t*-test), which is consistent with previous studies^37,38^. Gene expression analysis of DNA methylation-related factors based on the single-cell RNA-seq data showed that the expression of *Dnmt3l* was significantly elevated between the GO1 and GO2 stages (*P*-value = 1.8 × 10^−6^ between GO1 and GO2 stages, two-tailed Student’s *t*-test), whereas *Dnmt3a* and *Tet3* were expressed at high levels during this whole period (Supplementary information, Fig. S3a). However, the global DNA methylation level, ranging from 0.21 to 0.31 in the GO3 stage based on the 25th and 75th percentiles, was heterogeneous among individual oocytes when approaching full maturation (Fig. 1d).

**Fig. 2:**
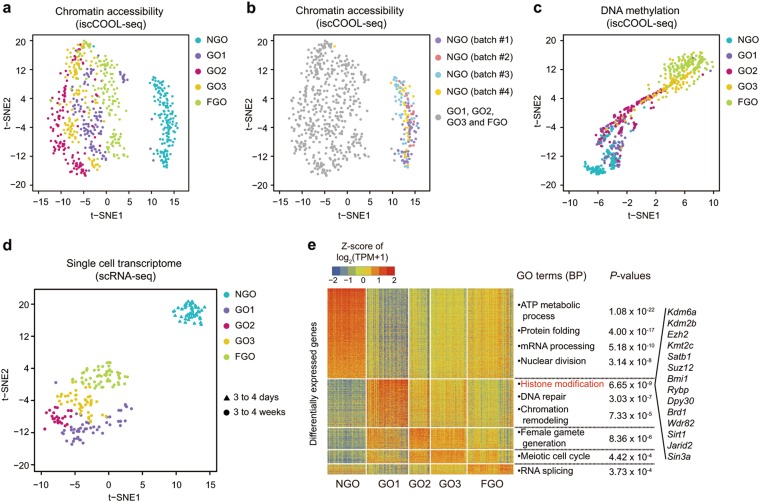
Integrative single-cell analysis of growing mouse oocytes. **a** t-SNE analysis of the chromatin accessibility (GCH methylation level) of growing mouse oocytes. **b** Batch effect analysis of the chromatin accessibility of NGO generated by iscCOOL-seq. **c **t-SNE analysis of the DNA methylation (WCG methylation level) of growing mouse oocytes. **d** t-SNE analysis of the single-cell RNA-seq data from growing mouse oocytes. **e** Analysis of the differentially expressed genes among single mouse oocytes

The most dramatic alteration in global chromatin accessibility occurred after the initiation of oocyte growth (Fig. 1e; Supplementary information, Fig. S2c), with median levels of 0.15 in the NGO stage and 0.21 in the GO1 stage (*P*-value = 2.0 × 10^−22^, two-tailed Student’s *t*-test). However, the accessibility of chromatin regions adjacent to TSSs (250 bp upstream and 100 bp downstream of the TSS) gradually decreased in individual oocytes during maturation (Fig. 1f). t-SNE analysis of chromatin accessibility of single oocytes also showed that the non-growing oocytes were clustered together and clearly separated from GOs and FGOs (Fig. 2a) with limited batch effect (Fig. 2b). However, the t-SNE analysis of DNA methylation of single oocytes showed successive changes from NGO to FGO stage (Fig. 2c).

The global pattern of gene expression also accorded with the change in chromatin accessibility (Fig. 2d, e; Supplementary information, Table S2). Although the number of active genes detected within each single oocyte was comparable at these stages, the relative mRNA abundance was significantly increased (*P*-value = 8.2 × 10^−18^, two-tailed Student’s *t*-test) at the GO1 stage compared to that at the NGO stage (Supplementary information, Fig. S3b, c). Moreover, there were 2,019 differentially expressed genes (DEGs) between the NGO and GO1 stages, which composed 74.80% of all DEGs (Fig. 2d, e; Supplementary information, Table S2). Additionally, Gene Ontology (GO) analysis showed that genes responsible for histone modification or chromatin remodelling were most enriched (*P*-value = 6.65 × 10^−9^ and 7.33 × 10^−5^, respectively, Fisher’s exact test) in the GO1 stage (Fig. 2e), indicating an important role for chromatin remodelling factors during oocyte growth. These results indicated that the non-growing oocytes were unique in gene expression and chromatin accessibility.

### Heterogeneity of chromatin accessibility in mouse GOs

**Fig. 3:**
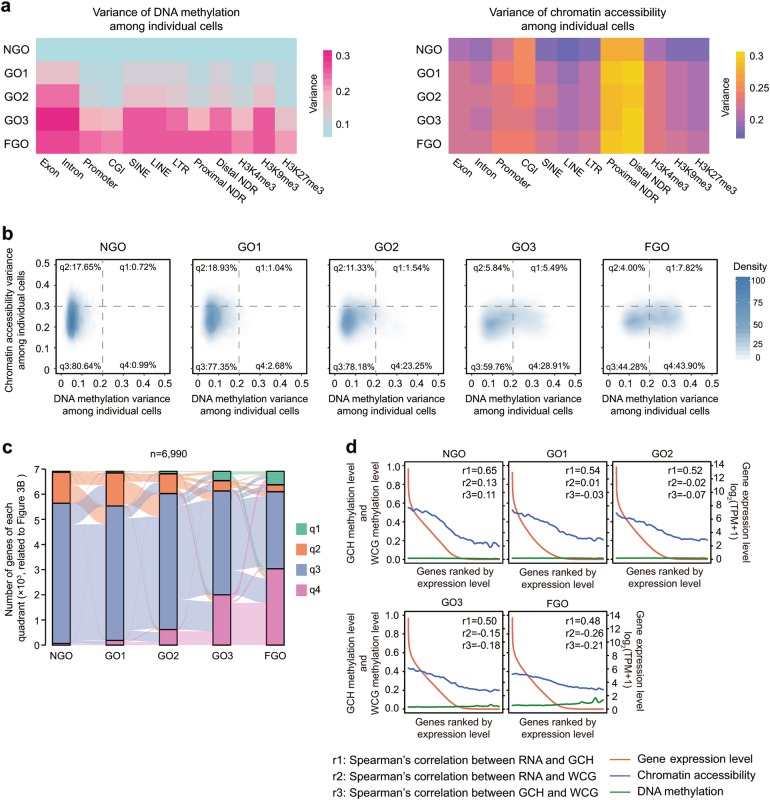
Single-cell variance in chromatin accessibility and DNA methylation during mouse oocyte maturation. **a** Median variance in DNA methylation and chromatin accessibility among individual cells in different genomic elements at each stage. **b** Density plots show the relationship between the variance in chromatin accessibility (200 bp upstream and 100 bp downstream of the TSS) and the variance in DNA methylation (1 kb upstream and 0.5 kb downstream of the TSS) in single oocytes. Only 6,990 genes covered at all stages were used to plot the density. The plots were separated to four quadrants: q1 indicates genes with high variance in both chromatin accessibility and DNA methylation; q2 indicates genes with high variance in chromatin accessibility but low variance in DNA methylation; q3 indicates genes with low variance in both chromatin accessibility and DNA methylation; q4 indicates genes with low variance in chromatin accessibility but high variance in DNA methylation. **c** Alluvial map showing the dynamics of genes in quadrants defined in Fig. 2b during mouse oocyte growth. Bars denote the number of genes in each quadrant at each stage, and lines between bars denote the number of genes transition from a certain quadrant at earlier stage to another quadrant at later stage. **d** Relationship among the chromatin accessibility (200 bp upstream and 100 bp downstream of the TSS), DNA methylation (1 kb upstream and 0.5 kb downstream of the TSS) of promoters and the expression of the corresponding genes. Genes on the x-axis were ranked by the gene expression level

After analysing the global dynamics of chromatin accessibility during oocyte growth, we next explored the variance in different genomic elements among individual oocytes within each stage. Moreover, we analysed both the chromatin accessibility and DNA methylation in different functional elements to dissect the contribution of each element in methylation alteration. The variation trends of either chromatin accessibility or DNA methylation observed during oocyte growth were generally similar among different elements (Supplementary information, Figs. S4-S5), except for the CpG islands (CGIs) and high-density CpG promoters, in which both features remained comparable among different stages (Supplementary information, Figs. S4-S5). However, the heterogeneity of chromatin accessibility and DNA methylation varied in each genomic elements during oocyte growth (Fig. 3a). For NDRs, we divided NDRs into two groups, proximal and distal NDRs. The distal NDRs varied the most in chromatin accessibility across the whole period (median variances were 0.29, 0.31, 0.31, 0.31 and 0.32 in NGO, GO1, GO2, GO3 and FGO, respectively), while repetitive elements (median variances were all above 0.10 from GO1 to FGO, including SINE, LINE and LTR) and H3K9me3-modified chromatin (median variances were all above 0.11 from GO1 to FGO) showed significant DNA methylation change after oocyte growth began (Fig. 3a). Genes with a high variance (≥0.3) in chromatin accessibility at their promoters tended to have a low variance (<0.2) in DNA methylation in the NGO and GO1 stages (Fig. 3b). However, a subset of genes developed a high variance in both chromatin accessibility (≥0.3) and DNA methylation (≥0.2) when oocytes approached maturation (Fig. 3b, c). The promoters showed less heterogeneity in both chromatin accessibility and DNA methylation compared to those in NDRs (Fig. 3a). Because the promoters could be classified into high-density CpG-, intermediate-density CpG-, low-density CpG- and CpG island-promoters, we analysed the correlation among chromatin accessibility, DNA methylation and gene expression in each type. Moreover, we also analysed their correlation among them between differentially expressed and non-differentially expressed genes during oocytes growth (Fig. 3d; Supplementary information, Fig. S6). Overall, the chromatin accessibility of gene promoters was positively correlated (calculated by the Spearman’s correlation) with the expression of the corresponding genes, while DNA methylation was weakly correlated (calculated by the Spearman’s correlation) with gene expression (Fig. 3d; Supplementary information, Fig. S6).

### Dynamic features of chromatin accessibility at imprinting control regions (ICRs), partially methylated domains (PMDs) and CGIs during oocyte growth

**Fig. 4:**
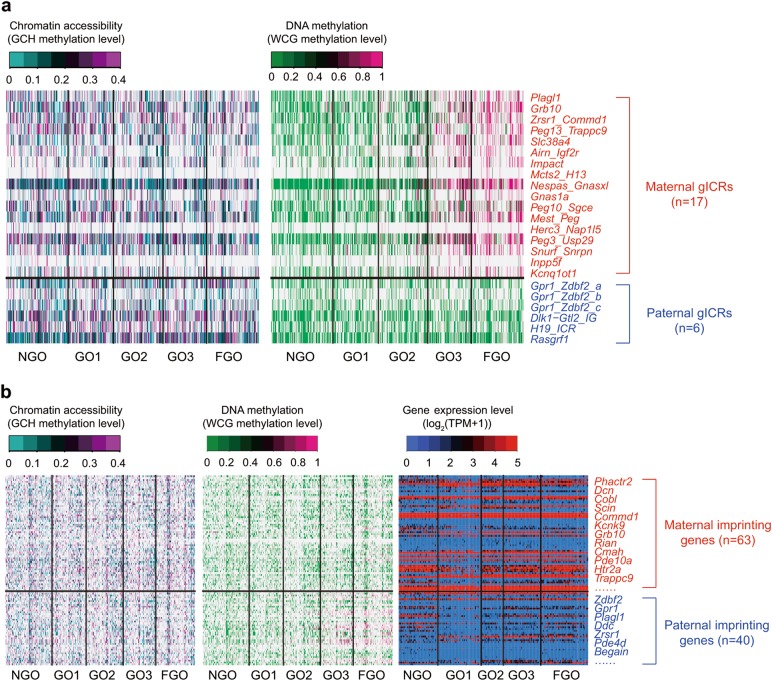
Dynamics of chromatin accessibility at germline imprinting control regions (gICRs) and imprinting genes during oocyte growth. **a** Heatmaps of chromatin accessibility and DNA methylation of gICRs at single-cell resolution. Maternal gICRs and paternal gICRs are labelled as red and blue colour, respectively. **b** Heatmaps of chromatin accessibility, DNA methylation and expression level of imprinting genes at single-cell resolution. Maternal imprinting genes and paternal imprinting genes are labelled as red and blue colour, respectively

The germline imprinting control regions (gICRs) of the maternal genome undergo de novo DNA methylation during oocyte growth^39,40^; however, the chromatin accessibility of these regions remained elusive. So, we analysed dynamics of both the chromatin accessibility and DNA methylation of gICRs at single-cell resolution. We found that the chromatin accessibility of maternal gICRs was generally comparable to that of paternal gICRs, despite the differences in DNA methylation between these two types of gICRs (Fig. 4a). Overall, maternal imprinting genes displayed similar accessibility at promoters with that of paternal imprinting genes, but showed less promoter DNA methylation and a higher level of expression (Fig. 4b).

**Fig. 5:**
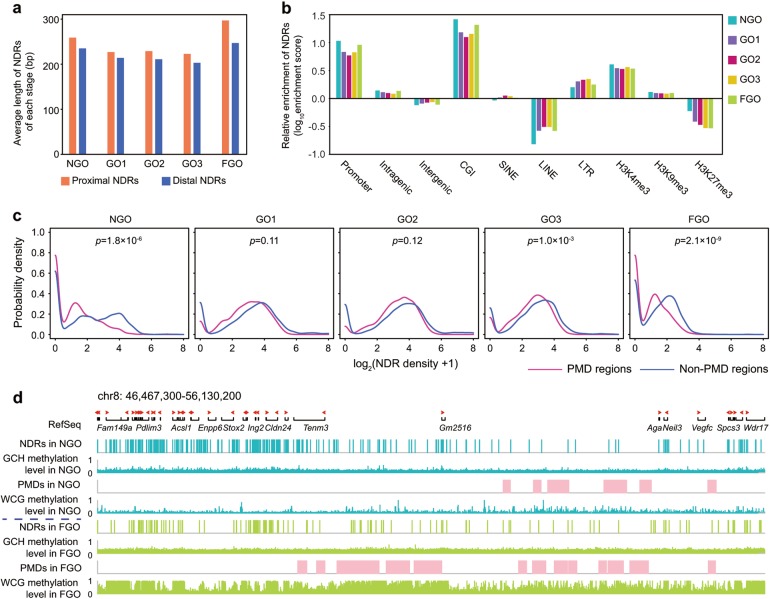
Probability density plots of NDR density in PMDs during oocyte growth. **a** Average length of proximal NDRs (within 2 kb upstream and downstream of the TSSs) and distal NDRs (at least 2 kb away from the TSSs) of each stage. **b** Relative enrichment of NDRs in different genomic elements during oocytes growth. **c** Probability density plots of NDR density in PMDs and non-PMDs in growing mouse oocytes. **d** Representative loci showing NDR density in PMDs and non-PMDs at NGO and FGO stage. The starting point and direction of red arrows at RefSeq panel represent the gene transcriptional starting site and gene transcriptional direction. *P*-values were defined by the two-tailed Student’s *t*-test

Next, we analysed the distribution of NDRs in different genomic elements within each stage. The lengths of NDRs (234 bp on average) generally changed little during oocytes growth (Fig. 5a). However, the NDRs were more enriched in CGIs (log_10_(enrichment score) = 1.42, 1.18, 1.10, 1.16, 1.32 in NGO, GO1, GO2, GO3 and FGO, respectively), promoters (log_10_(enrichment score) = 1.03, 0.83, 0.77, 0.82, 0.96 in NGO, GO1, GO2, GO3 and FGO, respectively), H3K4me3-modified chromatins (log_10_(enrichment score) = 0.61, 0.54, 0.53, 0.56, 0.53 in NGO, GO1, GO2, GO3 and FGO, respectively) and LTRs (log_10_(enrichment score) = 0.20, 0.30, 0.33, 0.35, 0.25 in NGO, GO1, GO2, GO3 and FGO, respectively) than the other elements analysed (Fig. 5b). Because PMDs and CGIs have been reported to be associated with H3K4me3 modifications in mouse oocytes, which is considered to be an active histone marker^41–43^, we next investigated the chromatin accessibility of these regions during oocyte growth. The density of the NDRs was comparable between the intergenic and intragenic regions within each stage (Supplementary information, Fig. S7a), so was that between the introns and exons (Supplementary information, Fig. S7b). However, the PMD regions had a significantly lower density of NDRs than the non-PMD regions in the NGO stage (*P*-value = 1.8 × 10^−6^, two-tailed Student’s *t*-test). NDR densities in these regions became comparable when oocyte growth began, despite that the density in PMD regions was still low (Fig. 5c, d; Supplementary information, Fig. S7c, d). Interestingly, the regions with high CpG densities, such as CGIs and HCPs, appeared to have higher NDR density during the NGO stage than the growth stages (Supplementary information, Fig. S7e; for CGI and HCP, *P*-values were all less than 0.05 between NGO and other stages, two-tailed Student’s *t*-test). Taken together, these findings indicated that although the PMDs and CGIs were both associated with H3K4me3 modifications, these genomic regions may be involved in different regulatory mechanisms of gene expression during oocyte growth.

### NDRs around TSSs and transcriptional end sites (TESs) during oocyte maturation

**Fig. 6:**
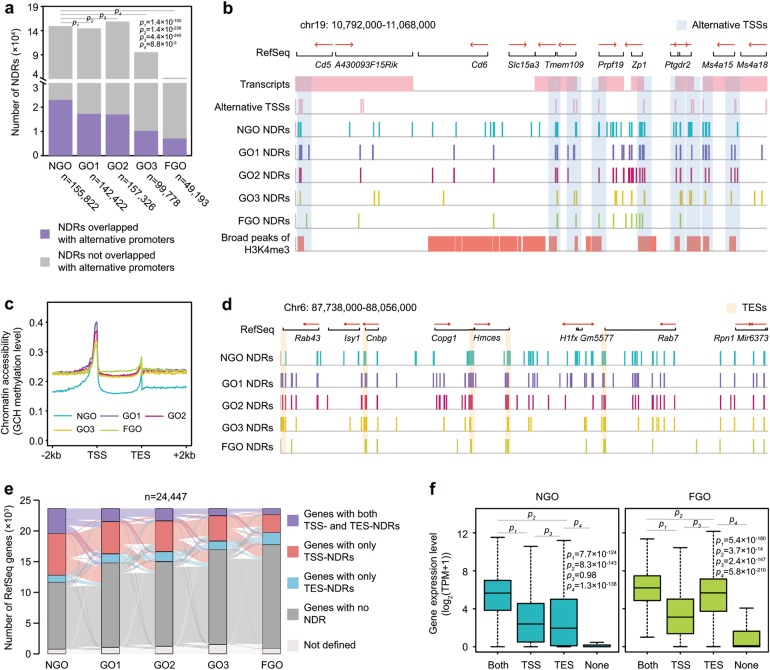
Dynamic features of NDRs at TSSs and TESs in growing mouse oocytes. **a** The number of total NDRs and NDRs overlapped with alternative promoters at each stage. P-values were defined by the chi square test. **b** Representative loci showing RefSeq genes, transcripts, alternative TSSs, NDRs and broad peaks of H3K4me3 at each stage. Alternative TSSs are labelled as blue shadows. The starting point and direction of red arrows at RefSeq panel represented the gene transcriptional starting site and gene transcriptional direction. **c** The average chromatin accessibility of RefSeq genes across gene bodies at each stage. **d** Representative loci showing gene TESs with NDRs. TESs are labelled with orange shadows. The starting point and direction of red arrows at RefSeq panel represent the gene transcriptional starting site and gene transcriptional direction. **e** Alluvial map showing the dynamics of genes with both TSS- and TES-NDRs, genes with only TSS-NDRs, genes with only TES-NDRs and genes with no NDR. TSS-NDRs and TES-NDRs were defined as NDRs overlapped with TSS regions (200 bp upstream and 100 bp downstream of the TSS) and TES regions (200 bp upstream and 100 bp downstream of the TES). Only genes with both covered TSS and TES regions (containing at least five GCH sites) were used. Bars denote the number of genes in each category at growing stage, and lines between bars denote the number of genes transition from a certain category at earlier stage to another category at later stage. **f** Expression levels of genes with both TSS and TES NDRs, genes with only TSS-NDRs, genes with only TES-NDRs and genes with no NDR at the NGO and FGO stage. P-values were defined by the two-tailed Student’s *t*-test

Because CpG-rich promoters have alternative TSSs in mouse oocytes^44^, they showed higher densities of NDRs in the NGO stage (Supplementary information, Fig. S7e) which was positively correlated with our gene expression data (Supplementary information, Fig. S6a; the Spearman’s correlation coefficients were all above 0.40 for HCP). We then analysed whether these alternative TSSs were associated with NDRs and found that nearly 40% of all the alternative TSSs (29,772 out of 76,899) identified in oocytes overlapped with NDRs in the NGO stage. However, this proportion decreased during oocyte maturation and reached the minimum in the FGO stage (Fig. 6a, b).

Next, we analysed the chromatin accessibility across whole gene bodies. Surprisingly, the regions adjacent to TESs were found to have relatively higher chromatin accessibility across all stages (Fig. 6c, d). Among all the genes studied, 17.20% had NDRs at both the TSS and TES in the NGO stage (Fig. 6e), however, in the FGO stage, the number decreased to 4.22% (Fig. 6e). 28.61% of genes had NDRs only at the TSS in the NGO stage, while only 12.32% were found to be open in the FGO stage (Fig. 6e). In contrast, 4.90% of the studied genes were found to have NDRs only at the TES in the NGO stage, but the number increased to 8.29% in the FGO stage (Fig. 6e). Taken together, these results indicated that different chromatin remodelling factors may be involved in the regulation of chromatin accessibility around TSSs and TESs, leading to an open-to-closed transition of the TSS during oocyte growth.

We then analysed gene expression. In the NGO stage, genes with NDRs at both the TSS and the TES were expressed at higher levels, while genes with no NDRs at either the TSS or the TES tended to be expressed at lower levels or were even silenced (Fig. 6f; Supplementary information, Fig. S8a). Interestingly, in the GO and FGO stages, genes with TES-NDRs were significantly more highly expressed (*P*-value = 8.2 × 10^−26^, 6.6 × 10^−41^, 3.0 × 10^−57^, 2.4 × 10^−147^ in GO1, GO2, GO3 and FGO, respectively, two-tailed Student’s *t*-test) than those with TSS-NDRs only (Fig. 6f; Supplementary information, Fig. S8a). This led us to analyse whether the NDRs at the TES played a role in transcriptional activation. Motif enrichment analysis showed that binding sites for the basic helix-loop-helix (bHLH) family of dimerizing transcription factors (TFs), such as Tcl12 (−log_10_(*P*-value) = 34, 30, 27, 24, 15 in NGO, GO1, GO2, GO3 and FGO, respectively), MyoD (−log_10_(*P*-value) = 33, 26, 23, 20, 16 in NGO, GO1, GO2, GO3 and FGO, respectively), E2A (−log_10_(*P*-value) = 30, 20, 23, 15, 10 in NGO, GO1, GO2, GO3 and FGO, respectively) and TfAp4 (−log_10_(*P*-value) = 25, 24, 22, 14, 13 in NGO, GO1, GO2, GO3 and FGO, respectively) were strongly enriched in these TES-NDRs (Supplementary information, Fig. S8b). This result indicated that gene loop structures, which have been found to be involved in gene transcription regulation in yeast^45^, may also play a role during mouse oocyte growth. Finally, we checked the dynamic expression of the group of genes with either TSS-NDR or TES-NDR and confirmed that genes with neither TSS-NDR nor TES-NDR were hardly expressed during oocytes growth (Supplementary information, Fig. S8c).

### Relationship among chromatin accessibility, de novo DNA methylation and gene expression in growing mouse oocytes

**Fig. 7:**
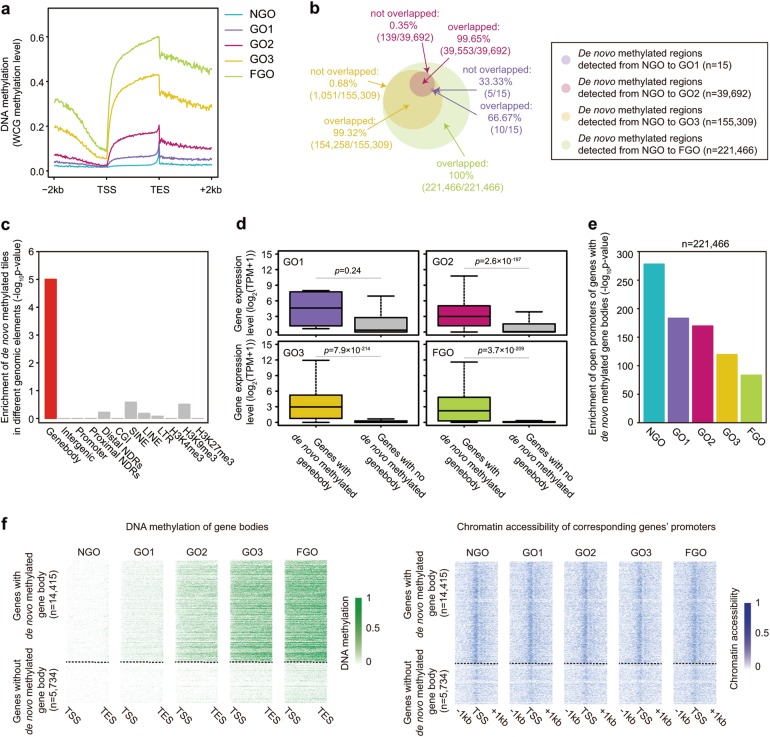
Relationship among chromatin accessibility, de novo DNA methylation and gene expression. **a** The average DNA methylation level of RefSeq genes across gene bodies at each stage. **b** Venn plot of de novo methylated regions detected from NGO to the later stages. **c** Enrichment of de novo methylated tiles detected from NGO to FGO in different genomic elements. *P*-values were calculated by the Fisher’s exact test. **d** Expression levels of genes with or without de novo methylated gene body. **e** The enrichment of open promoters at genes with de novo methylated gene body. Promoters (1 kb upstream and 0.5 kb downstream of the TSS) overlap with at least one NDR are defined as open promoters. *P*-values were calculated by the Fisher’s exact test. **f** Heatmap of DNA methylation of gene bodies and chromatin accessibility of corresponding genes’ promoters during oocyte growth. *P*-values were defined by the two-tailed Student’s *t*-test

De novo DNA methylation in mouse oocytes usually occurs within gene bodies and is associated with active transcription^37,38,44^. Our iscCOOL-seq data of mouse oocytes also confirmed this phenomenon (Fig. 7a-c; Supplementary information, Fig. S9a). However, little is known about the relationship between chromatin accessibility and de novo DNA methylation. We found that genes with either TSS-NDR or TES-NDR had higher DNA methylation levels in the gene bodies than genes with no NDRs at either the TSS or TES (Supplementary information, Fig. S9b; *P*-values were all less than 0.05, two-tailed Student’s *t*-test). Interestingly, genes with either TSS-NDR or TES-NDR also showed higher chromatin accessibility in the gene bodies than genes with no NDRs at either the TSS or TES (Supplementary information, Fig. S9b; *P*-values were all less than 0.05, two-tailed Student’s *t*-test). Furthermore, genes highly expressed were usually more accessible at the TSS or TES and had the highest DNA methylation levels in the gene bodies (Supplementary information, Fig. S9c). In contrast, genes with low expression levels were usually accessible only at the TSS and had moderate DNA methylation levels in the gene bodies (Supplementary information, Fig. S9c). Because genes with de novo methylated gene bodies were usually highly expressed in mouse oocytes (Fig. 7d), we next examined the chromatin accessibility of their promoters. Results showed that genes with de novo methylated gene bodies were significantly enriched (−log_10_(*P*-value) = 278, 183, 170, 120, 84 in NGO, GO1, GO2, GO3 and FGO, respectively by the Fisher’s exact test) with open promoters in mouse oocytes, as expected (Fig. 7e). Moreover, genes with de novo methylated gene bodies were already open at their promoters in the non-growing oocytes (Fig. 7f), whereas those without de novo methylated gene bodies were tended to be closed across the whole stages (Fig. 7f). As iscCOOL-seq could detect both chromatin accessibility and DNA methylation at single-cell level, we analysed the relationship between them within individual ES cells/oocytes across five stages. Different from mouse ES cells, weakly positive correlations for DNA methylation with chromatin accessibility were found in individual oocytes at both promoters and gene bodies (Supplementary information, Fig. S10; the median of weighted Pearson correlation coefficients were all above zero). Taken together, these results indicated that the chromatin openness, transcriptional activation and de novo DNA methylation were orchestrated during oocyte growth.

### Differences in chromatin accessibility between NGOs and GOs

**Fig. 8:**
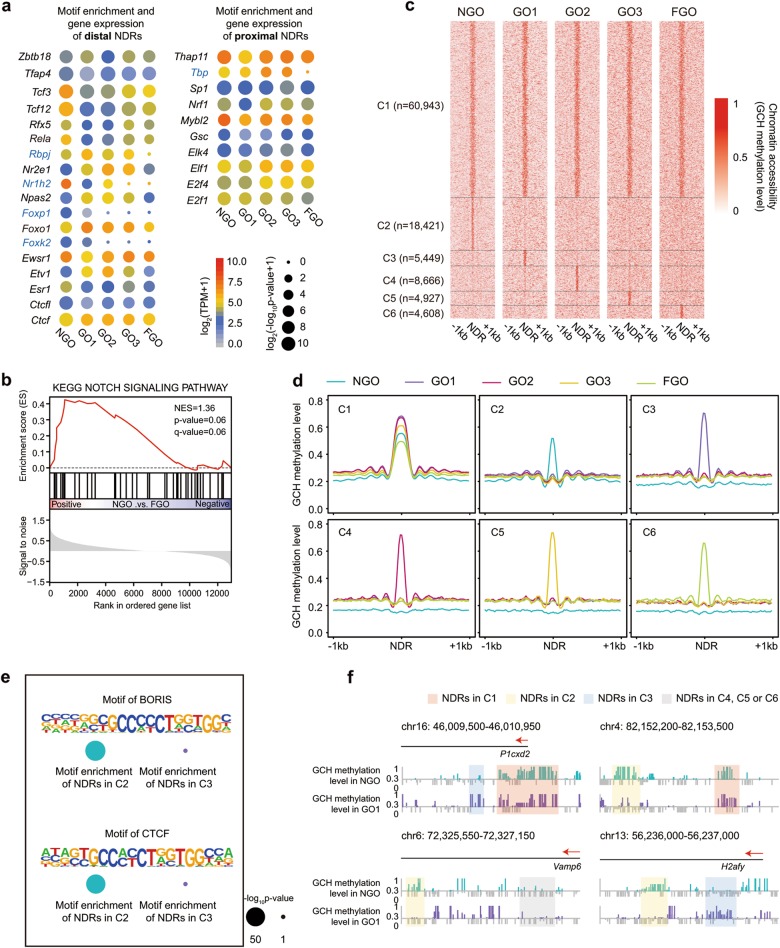
Dynamics of NDRs during mouse oocytes growth. **a** Motif enrichment analysis of distal NDRs (at least 2 kb away from the TSS, left panel) and proximal NDRs (within 2 kb upstream and downstream of the TSS, right panel) in growing mouse oocytes. Blue colour indicates stage specific motifs. **b** GSEA analysis of Notch signalling pathway-related genes between NGOs and FGOs. The scRNA-seq data was used here. **c** Stage specific analysis of NDRs during mouse oocytes growth. Cluster 1 (C1) indicates NDRs detected in all stages; Cluster 2 (C2) indicates NDRs detected only in NGO stage; Cluster 3 (C3) indicates NDRs detected only in GO1 stage; Cluster 4 (C4) indicates NDRs detected only in GO2 stage; Cluster 5 (C5) indicates NDRs detected only in GO3 stage; Cluster 6 (C6) indicates NDRs detected only in FGO stage. **d** Chromatin accessibility around NDRs (±1 kb) in growing mouse oocytes. C1 to C6 are defined as same as those in Fig. 8c. **e** Comparison of CTCF/BORIS binding motif enrichment between NDRs in C2 (NGO specific NDRs) and C3 (GO1 specific NDRs). **f** Representative loci showing NDRs defined in Fig. 8c

Because NGOs showed differences from GOs and FGO in chromatin accessibility and gene expression (Fig. 2), we further tried to analyse and identify TFs that may be involved in the regulation of oocyte maturation. Firstly, we performed motif enrichment analysis. We divided NDRs into two groups, proximal and distal NDRs, and found that the majority of TFs showed comparable enrichment scores across the whole maturation period and are known to act as constitutive TFs, such as E2f1, E2f4, Elf1 and Ctcf (Fig. 8a). Some TFs, including Tbp (log_2_[−log_10_(*P*-value) + 1] = 4.8, 4.2, 4.0, 3.7, 0.0 in NGO, GO1, GO2, GO3 and FGO, respectively), Rbpj (log_2_[−log_10_(*P*-value) + 1] = 4.8, 5.9, 6.1, 4.8, 0.0 in NGO, GO1, GO2, GO3 and FGO, respectively), Nr1h2 (log_2_[−log_10_(*P*-value) + 1] = 4.4, 4.4, 3.7, 0.0, 0.0 in NGO, GO1, GO2, GO3 and FGO, respectively), Foxp1 (log_2_[−log_10_(*P*-value) + 1] = 5.9, 3.8, 0.0, 0.0, 0.0 in NGO, GO1, GO2, GO3 and FGO, respectively) and Foxk2 (log_2_[−log_10_(*P*-value) + 1] = 5.8, 3.8, 0.0, 0.0, 0.0 in NGO, GO1, GO2, GO3 and FGO, respectively), showed stage-specific enrichment in NGOs and early GOs (Fig. 8a). Furthermore, we analysed potentially important TFs through SCENIC algorithm^46^ by using scRNA-seq data of individual oocytes (Supplementary information, Fig. S11a). Consistent with the results of motif enrichment analysis, Foxp1 was also identified by SCENIC algorithm in the NGO and GO1 stages (Supplementary information, Fig. S11a), suggesting its role in early oocytes growth.

Interestingly, binding motif of Rbpj, which is a downstream TF of the Notch signalling pathway, showed significant enrichment (log_2_[−log_10_(*P*-value) + 1] = 4.8, 5.9, 6.1, 4.8, 0.0 in NGO, GO1, GO2, GO3 and FGO, respectively) in the NGO and GO stages (Fig. 8a). When we performed a gene set enrichment analysis (GSEA), we found a relative enrichment (Normalized Enrichment Score = 1.36, *P*-value = 0.06) of Notch signalling pathway-related genes in NGOs compared to FGOs (Fig. 8b). Moreover, many genes involved in the Notch signalling pathway were expressed during oocyte maturation (Supplementary information, Fig. S11b). These results suggested that Notch signalling pathway might be involved in oocyte maturation. Therefore, we took use of an in vitro culture system of mouse follicles to verify the role of Notch signalling pathway in oocytes growth. After treated with small molecule inhibitors targeted to Notch signalling pathway, the in vitro maturation of follicles was compromised, indicating an important role of Notch signalling during oocytes growth (Supplementary information, Fig. S11c, d).

Finally, we performed a differential NDR analysis and found that 18,421 of NDRs detected in the NGO stage became closed after oocyte growth initiation (Fig. 8c, d). These NDRs were more enriched with binding motifs for CTCF (−log_10_(*P*-value) = 58, 1 in NGO and GO1) and BORIS, also known as CTCFL (−log_10_(*P*-value) = 52, 1 in NGO and GO1) (Fig. 8e, f), which are two paralogous proteins that serve as global organizers of chromatin architecture and play important roles in the regulation of gene transcription^47^. Taken together, these results indicated that oocyte growth was a complex but highly ordered process.

## Discussion

In this study, we developed an improved single-cell COOL-seq method, iscCOOL-seq, based on the TAILS strategy (Supplementary information, Fig. S1a), which achieved a mean alignment rate of 62.26% in single cells (Supplementary information, Fig. 1b). This mapping rate is nearly twofold higher than that achieved with our previously reported scCOOL-seq by the PBAT strategy^29^. Compared to the previous PBAT method, TAILS, which is a strategy different from the sci-MET^34^ or snmC-seq^33^ methods, simplifies the steps in constructing single-cell DNA methylome libraries and reduces the sequencing costs. By using the iscCOOL-seq method together with scRNA-seq, for the first time, chromatin accessibility, DNA methylation and gene expression of growing mouse oocytes were integrated and analysed at single-cell and single-base resolution.

We found that the most dramatic change in chromatin accessibility happened during NGO to GO transition (Fig. 1e; Supplementary information, Fig. S2c), with prominent changes in the transcriptome (Fig. 2d, e) and an elevated variation in DNA methylation at H3K9me3-modified chromatins (Fig. 3a) among individual oocytes. Recent studies have identified H3K9me3 modification as a key epigenetic barrier to somatic cell nuclear transfer (SCNT) in mouse, monkey and human^48–50^. The impact of H3K9me3 modification during oocyte growth needs further study.

We found that the PMDs, which have been associated with non-canonically broad H3K4me3 peaks in oocytes^41^, displayed low density of NDRs during oocyte maturation (Fig. 5c, d). Previous works have reported that aberrant H3K4 methylation disrupts de novo DNA methylation in maternal imprinting^51^, and the removal of H3K4 methylation by Kdm1a/1b is necessary to establish proper DNA methylation at these imprinted CGIs^36^. Deficiency in genes that modulate H3K4 methylation, such as Cfp1 or Sall4, prevents oocyte maturation^52,53^. The non-canonical accumulation of H3K4me3 in growing mouse oocytes^41,43^ has been associated with poor transcriptional activation. Furthermore, Mll2, which antagonizes Dnmt3a/3b, conveys transcription-independent H3K4me3 in mouse oocytes^20^. Therefore, it will be interesting to investigate the alteration of chromatin accessibility at PMDs in Mll2-, Kdm1a/1b- or Dnmt3a/3b-deficient oocytes in the future.

We found that a proportion of genes harboured both TSS- and TES-NDRs in NGOs. However, genes tended to be closed or harbour only TES-NDRs in FGOs (Fig. 6e; Supplementary information, Fig. S8). Furthermore, these TES-NDRs were strongly enriched with binding sites for the basic helix-loop-helix (bHLH) family of dimerizing TFs (Supplementary information, Fig. S8b). This result indicated that gene loop structures, which have been found to facilitate gene transcription in yeast^45^, may also play a role during mouse oocyte growth. It will be better to perform ChIA-PET against RNA PolII in oocytes to specify this feature in future studies.

By performing a differential gene expression and a motif enrichment analysis, we found that some epigenetic and transcription factors showed stage-specific enrichment during oocyte maturation and so did certain genes involved in the Notch signalling pathway were expressed (Figs. 2e, 8a and 8b; Supplementary information, Fig. S11). Additionally, CTCF and BORIS (CTCFL) binding motifs were found to be enriched in specific NDRs detected in NGOs (Fig. 8e). The specific roles of these factors in mouse oocyte growth warrant further studies.

In summary, we used iscCOOL-seq together with scRNA-seq, for the first time, to perform an integrated analysis of chromatin accessibility, DNA methylation and gene expression in growing mouse oocytes at single-cell and single-base resolution. Our work paves the way for dissecting the complex, yet highly coordinated, epigenetic alterations during mouse oocyte maturation and the establishment of totipotency.

## Materials and methods

### Isolation, in vitro culture of mouse follicles and collection of growing mouse oocytes

All animal procedures were carried out according to the ethical guidelines of the Laboratory Animal Center of Sichuan University. To collect mouse follicles for in vitro culture^54^, ovaries were obtained from 14-day-old female ICR mice and transformed into L15 medium (Leibovitz, Gibco #11415064), which supplemented with 10% of FBS (foetal bovine serum, Vistec #SE200-ES) and 1 ×penicillin-streptomycin (Gibco #15140122). Then the ovaries were mechanically dissected and treated with 1 mg/mL of collagenase IV (Gibco #17104019) at 37 °C for 20 min. Released intact follicles with diameters around 120 μm were manually picked and cultured individually in 20-μL culture medium: α-MEM (Gibco #12561056) supplemented with 5% of FBS, 1 × ITS liquid media supplement (Sigma #I3146), 1 × of nucleosides (Millipore #ES-008-D), 1 × penicillin-streptomycin and 100 mIU/mL of recombinant human FSH (Merck Serono #Gonal-f). Follicles were cultured in the incubator at 37 °C and 5% CO_2_ in air and 10 μL of culture medium was added to each follicle after 24 h. The refreshment was performed every other day by replacing 10 μL of culture medium for individual follicles.

For inhibition of the Notch signalling pathway during in vitro follicles growth, small molecules targeted to Notch signalling were added into the follicles culture medium (25 μM in final concentration); these inhibitors were DAPT (Selleck #S2215), RO-4929097 (Selleck #S1575) and YO-01027 (Selleck #S2711), respectively. For each replicate, experiments for control and three of inhibitor groups were conducted in parallel. A total of 364 follicles from 4 replicated experiments were analysed, and the number of follicles for each group was: *n* = 93 for control group, *n* = 89 for DAPT group, *n* = 89 for RO-4929097 group, *n* = 93 for YO-01027 group.

To collect mouse oocytes, dissected ovary tissues, which were treated with collagenase IV, were further treated with TrypLE Express enzyme (Gibco #12604021) at 37 °C for 20 min to release the oocytes. Non-growing oocytes (NGOs) with diameters less than 20 μm were collected from 3- to 4-day-old female ICR mice. Growing oocytes (GOs) and fully-grown oocytes (FGOs) were collected from 3- to 4-week-old female ICR mice; the diameters of oocytes at each stage were as follows: GO1 (30 μm to <45 μm), GO2 (50 μm to < 55 μm), GO3 (60 μm to < 65 μm), and FGO (>70 μm). Excessive washes were performed to remove the contamination of somatic cells from the oocytes. Individual oocytes were then picked for the downstream single-cell experiments.

### Improved single-cell COOL-seq (iscCOOL-seq) library construction

First, individual oocytes were methylated in vitro by GpC methylase (New England Biolabs), digested by protease and bisulfite converted as previously described^29,30^. Then, the single-cell genomic DNA was purified and subjected to iscCOOL-seq library construction by TAILS method. Briefly, a round of random amplification was performed by tagging the P5-N6-oligo1 (5′-CTACACGACGCTCTTCCGATCTN_6_-3′) in the presence of 1 × Blue buffer (Enzymatics), 600 nM of dNTPs, 400 nM of P5-N6-oligo1 and 50 units of Klenow exo^−^ (Enzymatics) and incubated at 37 °C for 30 min. Then, the remained oligos and dNTPs were deactivated by Exo-SAP IT reagent (Applied Biosystems) following the manufacture’s protocol. Next, dC tailing was conducted in the presence of 1 × Blue buffer (Enzymatics), 2.5 mM of dCTPs and 1U/μL of TdT enzyme. After that, the second strand synthesis was performed by tagging the P7-G6-oligo2 (5′-AGACGTGTGCTCTTCCGATCTG_6_HN-3′) in the presence of 1 × Blue buffer (Enzymatics), 600 nM of dNTPs, 400 nM of P7-G6-oligo2 and 100 units of Klenow exo^−^ (Enzymatics) and incubated at 37 °C for 1 h, followed by Agencourt AMPure XP beads (Beckman) purification. Final libraries were generated by eighteen cycles of PCR to incorporate universal primers and index primers (New England Biolabs), and purified by Agencourt AMPure XP beads. The libraries were pooled, checked and sequenced with paired-end 150-bp reads on an Illumina HiSeq X-Ten platform (Novogene).

### Single-cell RNA-seq library construction

First, individual mouse oocytes were manually picked, lysed and subjected to first-strand cDNA synthesis as reported previously^8,9,29^. Then, second-strand cDNA was synthesized, amplified and fragmented. An RNA-seq library was prepared by following the instruction manual of the KAPA Hyper Prep Kit (KAPA Biosystems). Finally, the libraries were checked, pooled together and sequenced with paired-end 150-bp reads on an Illumina HiSeq X-Ten platform (Novogene).

### Processing the bioinformatic data

#### Data quality control and alignment of the iscCOOL-seq data

The data quality was checked and reads were mapped according to the protocols in our recent publication^29^. Briefly, Trim Galore (v0.4.4) was used to remove bases of random primer sequences, adaptor sequences and low-quality bases. Reads shorter than 50 bp after trimming were excluded. Bismark (v0.7.6) was adopted to align clean reads to the mouse reference genome mm9 by paired-end mode. Then, unmapped reads were realigned to mm9 by single-end mode. PCR duplicates were removed by SAMtools (v0.1.18) and only non-duplicated reads were retained for further analysis.

#### Determination of WCG and GCH methylation levels

The WCG and GCH methylation levels in individual oocytes were calculated as previously reported^29^. Briefly, the WCG (W denotes A or T) and GCH sites (H denotes A, C or T) with at least 1 × sequencing depth in each individual oocyte were summed. Then, the DNA methylation level and chromatin accessibility of the oocytes were estimated as the average WCG or GCH level, respectively.

#### Genomic region annotations

The annotations of CpG islands (CGIs), exons, introns, transcription start sites (TSSs) and transcription end sites (TESs) were downloaded from the UCSC genome browser (mm9). Intragenic regions and gene bodies were defined as the regions from TSS to TES, while intergenic regions were the complementary regions to the intragenic regions in mouse genome. Promoters were defined as the 1 kb upstream and 0.5 kb downstream of TSSs and can be classified into high-density CpG promoters (HCP), intermediate-density CpG promoters (ICP) and low-density CpG promoters (LCP) as previous study^29^. All the information about repetitive elements were obtained from the mm9 Repeat Masker. Germline imprinting control regions (gICRs) and imprinting genes were obtained from previous study^23^.

#### Definition of NDRs

To define the NDRs at each stage, the aggregated iscCOOL-seq data were used, and 120-bp sliding windows with 20-bp spacing were tested for differences from the genomic background using the χ^2^ test. The sliding windows with *P* values less than 10^−10^ were retained and were overlapped with each other. A region was finally defined as an NDR if it was a minimum of 140 bp in size and covered at least five GCH sites^29^.

#### Definition of NDR density

For a certain functional region, the NDR density was defined as follows: The lengths of NDRs within this functional region were added in unit of bp, then the summation was divided to length of this functional region in unit of kb.

#### Calculation of PMDs and non-PMDs in growing mouse oocytes

Partially methylated domains (PMDs) were calculated by the MethylSeekR package in R. Promoters (1 kb upstream and 0.5 kb downstream of the TSSs) were excluded from PMDs. Only PMDs more than 100 kb were retained. Non-PMDs were the regions complementary to PMDs in mouse genome.

#### Calculation of the variance in DNA methylation and chromatin accessibility

To calculate the variance in DNA methylation among single oocytes, the genome was divided into 3000-bp sliding windows with 600-bp steps. For each single cell, only windows covering more than 3 WCG sites were retained for further analysis. Then, the average methylation level of each retained window in every individual cell was calculated. Only windows covered in at least 10% of the single cells in each GO stage were evaluated. Thus, for windows passed the criteria, a previous reported^29^ and gold-standard method of methylation level were calculated among single cells at the same stage^30^. The variance in chromatin accessibility was estimated analogously. The genome was divided into 200-bp sliding windows with 100-bp steps. For each single cell, only windows covering more than 5 GCH sites were retained for further analysis. Then, the average methylation level of each retained window in every individual cell was calculated. Only windows covered in at least 10% of the single cells in each GO stage were evaluated. Thus, for windows passed the criteria, variances of chromatin accessibility were calculated among single cells at the same stage. To analyse the relationship between the variances in DNA methylation (1 kb upstream and 0.5 kb downstream of the TSS) and chromatin accessibility (0.2 kb upstream and 0.1 kb downstream of the TSS), density plots were generated^29^.

#### Analysis of de novo methylated regions

The de novo methylated regions were identified based on the following criteria: Firstly, we extracted the WCG sites which were covered in at least three single cells within each stage. Then, genome was divided into 1-kb tiles. Only tiles covering at least five WCG sites were retained to calculate the average methylation levels. A tile at NGO stage with at least a 0.15 average methylation level increase in GO1, GO2, GO3 or FGO stages (Benjamini-Hochberg’s FDR < 0.05) was defined as de novo methylated in GO1, GO2, GO3 or FGO. According to this cutoff, we identified 15 methylated tiles, 39,692 methylated tiles, 155,309 and 221,466 methylated tiles in GO1, GO2, GO3 and FGO, respectively.

#### Analysis of correlation between WCG and GCH in individual cell

The correlation between DNA methylation and chromatin accessibility at different genomic regions in each single cell was calculated based on the following criteria: Only genomic regions covering at least 3 WCG and 5 GCH sites in the same single cell were retained. A minimum number of 20 cells were required to calculate a correlation. Considering variant coverage between cells, we used a weighted Pearson correlation coefficient as previous studies^32^, and the coverage of WCG sites were used as a weight.

#### Identification of stage specific NDRs during oocyte maturation

Firstly, NDRs detected in aggregated single oocytes from each stage were merged together. Then, the GCH methylation levels of these regions were tested for differences against the genomic background by using Wilcoxon rank sum test. If regions covering at least five GCH sites and had significantly high GCH methylation level (*P*-value < 0.05), these regions were defined as open; If regions covering at least five GCH sites but had no significance in GCH methylation level (*P*-value ≥ 0.05), these regions were defined as closed; If regions were not covered in a specific stage (less than five GCH sites covered), these regions were not defined in this stage.

#### t-SNE analysis of chromatin accessibility, DNA methylation and gene expression level

The cell population was visualized by t-SNE algorithm which is a nonlinear dimensionality reduction technique. For the t-SNE analysis of chromatin accessibility, stage-specific NDRs were used. The GCH methylation levels of these stage-specific NDRs in individual cells were tested for differences against the genomic background by using Wilcoxon rank sum test. If a region covering at least five GCH sites in a single cell and has significantly high GCH methylation level (*P*-value < 0.05), this region was defined as open (labelled as “1”) in this single cell; If a region covered by at least five GCH sites in a single cell and had no significance in GCH methylation level (*P*-value ≥ 0.05), this region was defined as closed (labelled as “0”) in this single cell; if a region was not covered in a single cell (less than five GCH sites covered), this region was not defined (labelled as “NA”) in this single cell. Thus, the accessibility of each specific NDR within an individual cell can be transformed to a binary vector. Then the “Rand” indexes were calculated between each two single cells and the final similarity matrix was used to perform the t-SNE analysis of the single-cell chromatin accessibility.

For the t-SNE analysis of DNA methylation, the genome was divided into 50-kb windows and their average WCG methylation levels in each individual cell were calculated. The matrix of average DNA methylation level was used to perform the t-SNE analysis. For the t-SNE analysis of gene expression level, the expression level of highly variable genes identified by Seurat package in R were used. All the above t-SNE analyses used the Rtsne package in R.

#### Analysis of motif enrichment in NDRs

The HOMER v4.9.1 software was used to analyse motif enrichment in NDRs defined in mouse oocytes. Motifs with P values less than 10^−12^ were considered significantly enriched in the indicated stages.

#### Processing the single-cell RNA-seq data

Raw reads were first classified by cell barcodes, then trimmed and aligned (Tophat v2.0.12) to the mm9 mouse transcriptome. After de-duplication based on UMI information, the transcript copy numbers of each gene were counted^55^. Individual cells with more than 3000 detected genes, and transcripts between 20,000 and 4,000,000 were retained for further analysis. Then, individual cells with high ratio of ERCC spike-ins (with counts that were more than 5 median absolute deviations above the median) were removed (scater R package). After rigorous filtration, 223 out of 296 single cells with high quality were retained and used for the downstream analysis with the Seurat R package^56^, including identification of differentially expressed genes (DEGs). The “clusterProfiler” package in R was adopted to perform the GO analysis. Gene-set enrichment analysis (GSEA) was conducted following the recommended process. For each stage, the number of oocytes used for final analysis was: *n* = 46 for NGO, *n* = 51 for GO1, *n* = 26 for GO2, *n* = 44 for GO3 and *n* = 56 for FGO.

#### Statistical analysis

Each box represented the quartiles. The line inside the box was the second quartile (the median). The ends of the whiskers represented the lowest datum still within 1.5 IQR of the lower quartile and the highest datum still within 1.5 IQR of the upper quartile. Z-scores were calculated as:

$$z = \frac{{x - {\mathrm{\mu }}}}{\sigma }$$

where *µ* is the mean of the population, *σ* is the standard deviation of the population.

#### Bioinformatic code availability

The bioinformatic analysis was performed based on published algorithms with our custom codes, which are available upon reasonable request.

### Publicly available data sets used in this study

The published datasets used in this study were downloaded from the NCBI Gene Expression Omnibus (GEO) with the following accession numbers: GSE78140 (single-cell COOL-seq by the PBAT strategy and the PBAT method), PRJNA397747 (the sci-MET method), GSE97179 (the snmC-seq method), GSE86297 (WGBS of mouse GO3-stage oocytes using the PBAT method), GSE74549 (WGBS of mouse FGO-stage oocytes using the PBAT method), GSE70116 (whole-transcriptome profiling of mouse oocytes), GSE71434 (H3K4me3 ChIP-seq of mouse oocytes), GSE97778 (H3K9me3 ChIP-seq of mouse oocytes), GSE76687 (H3K27me3 ChIP-seq of mouse oocytes), GSE37074 (DNase-seq of mouse ES cells), GSE56879 (scBS-seq of mouse ES cells and MII oocytes), GSE29184 (H3K4me3 ChIP-seq of mouse ES cells), GSE62380 (H3K9me3 and H3K27me3 ChIP-seq of mouse ES cells).

### Data availability

All the sequencing data generated in this study, including iscCOOL-seq data and scRNA-seq data, were deposited in the NCBI Gene Expression Omnibus (GEO) under the accession number GSE114822. The authors declare that all the data supporting the findings of this study are available from the corresponding author on reasonable request.

## Electronic supplementary material

Supplementary information, Figure S1

Supplementary information, Figure S2

Supplementary information, Figure S3

Supplementary information, Figure S4

Supplementary information, Figure S5

Supplementary information, Figure S6

Supplementary information, Figure S7

Supplementary information, Figure S8

Supplementary information, Figure S9

Supplementary information, Figure S10

Supplementary information, Figure S11

Supplementary information, Table S1

Supplementary information, Table S2
