# Supplementary material for: Integrative single-cell analysis of transcriptome, DNA methylome and chromatin accessibility in mouse oocytes
Source: Cell Res. 2018 Dec 18;29(2):110–23. doi: 10.1038/s41422-018-0125-4 (PMC6355938; doi:10.1038/s41422-018-0125-4)
Supplement: Supplementary file 1 — Supplementary information, Figure S1 [file 41422_2018_125_MOESM1_ESM.pdf]

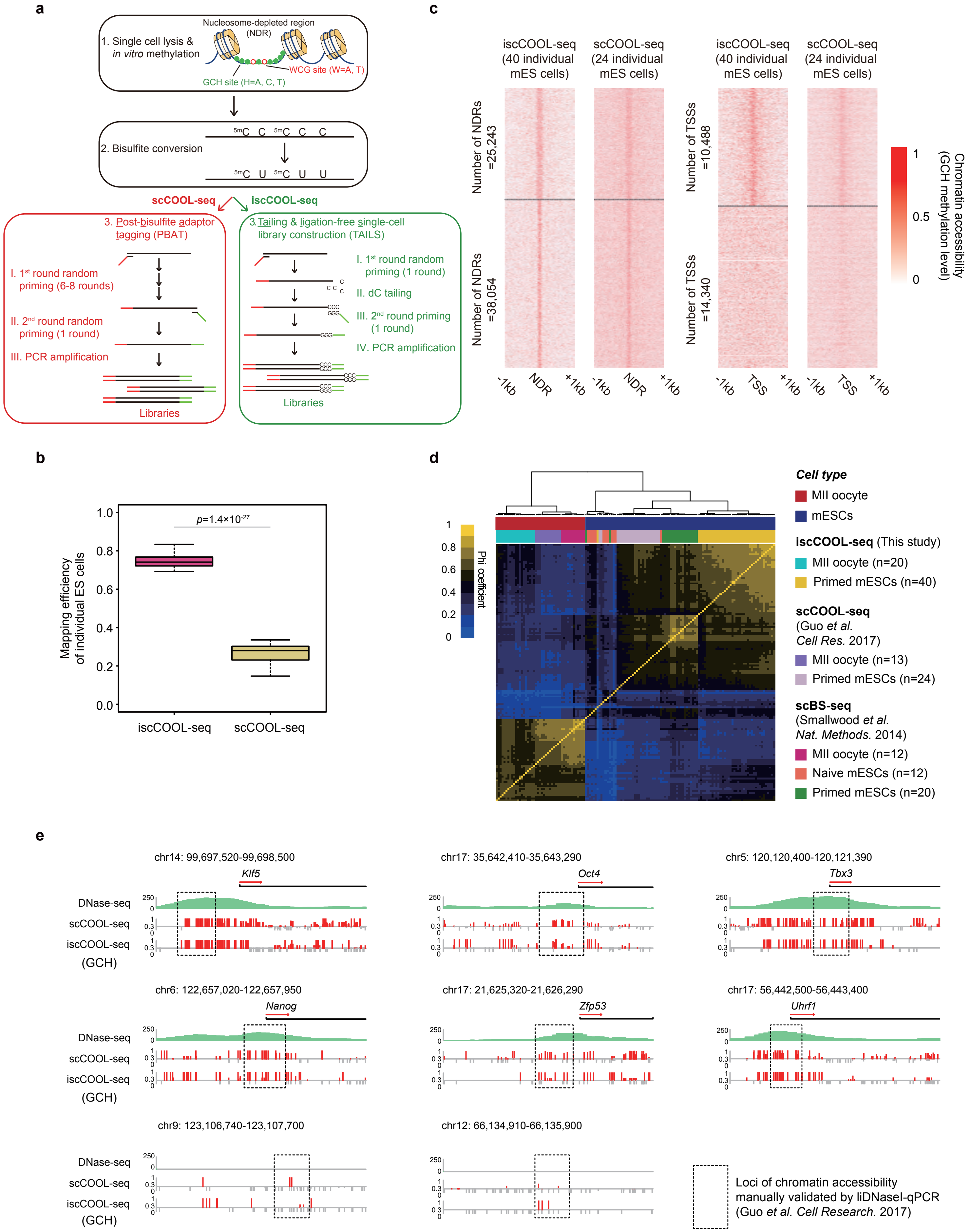
a
Nucleosome-depleted region (NDR)
1. Single cell lysis & in vitro methylation
GCH site (H=A, C, T)
WCG site (W=A, T)
2. Bisulfite conversion
scCOOL-seq
iscCOOL-seq
3. Post-bisulfite adaptor tagging (PBAT)
I. 1st round random priming (6-8 rounds)
II. 2nd round random priming (1 round)
III. PCR amplification
Libraries
3. Tailing & ligation-free single-cell library construction (TAILS)
I. 1st round random priming (1 round)
II. dC tailing
III. 2nd round priming (1 round)
IV. PCR amplification
Libraries
b
Mapping efficiency of individual ES cells
p=1.4×10-27
iscCOOL-seq
scCOOL-seq
c
iscCOOL-seq (40 individual mES cells)
scCOOL-seq (24 individual mES cells)
Number of NDRs =25,243
Number of NDRs =38,054
Number of TSSs =10,488
Number of TSSs =14,340
-1kb NDR +1kb
-1kb TSS +1kb
Chromatin accessibility (GCH methylation level)
d
Phi coefficient
Cell type
MII oocyte
mESCs
iscCOOL-seq (This study)
MII oocyte (n=20)
Primed mESCs (n=40)
scCOOL-seq (Guo et al. Cell Res. 2017)
MII oocyte (n=13)
Primed mESCs (n=24)
scBS-seq (Smallwood et al. Nat. Methods. 2014)
MII oocyte (n=12)
Naive mESCs (n=12)
Primed mESCs (n=20)
e
chr14: 99,697,520-99,698,500
Klf5
chr17: 35,642,410-35,643,290
Oct4
chr5: 120,120,400-120,121,390
Tbx3
chr6: 122,657,020-122,657,950
Nanog
chr17: 21,625,320-21,626,290
Zfp53
chr17: 56,442,500-56,443,400
Uhrf1
chr9: 123,106,740-123,107,700
chr12: 66,134,910-66,135,900
DNase-seq
scCOOL-seq
iscCOOL-seq
(GCH)
Loci of chromatin accessibility manually validated by liDNaseI-qPCR (Guo et al. Cell Research. 2017)

Supplementary information, Fig. S1 Sensitivity and accuracy of the iscCOOL-seq method. **(a)** Diagram of the improved single-cell COOL-seq method by using the TAILS strategy. **(b)** Comparison of mapping efficiency of individual mESCs between iscCOOL-seq and scCOOL-seq. **(c)** A heatmap showed that both the chromatin accessibility of regions around NDRs (± 1kb) and TSSs (± 1kb) detected by scCOOL-seq in mouse ESCs can be reproduced in iscCOOL-seq. **(d)** A heatmap showed the phi coefficients of digital DNA methylation level of individual mouse MII oocytes and ES cells performed by iscCOOL-seq, scCOOL-seq (Guo *et al. Cell Research*. 2017) or scBS-seq (Smallwood *et al. Nature Methods*. 2014). **(e)** Representative loci showed that the open regions (*Klf5*, *Oct4*, *Tbx3*, *Nanog*, *Zfp53* and *Uhrf1*) and closed regions (chr9: 123,106,740-123,107,700 and chr12: 66,134,910-66,135,900) in mouse ES cells, confirmed by liDNaseI-qPCR and scCOOL-seq (Guo *et al. Cell Research*. 2017), could be reproduced by iscCOOL-seq. P-values were defined by the two-tailed Student's *t*-test.
