# Supplementary material for: Integrative single-cell analysis of transcriptome, DNA methylome and chromatin accessibility in mouse oocytes
Source: Cell Res. 2018 Dec 18;29(2):110–23. doi: 10.1038/s41422-018-0125-4 (PMC6355938; doi:10.1038/s41422-018-0125-4)
Supplement: Supplementary file 2 — Supplementary information, Figure S2 [file 41422_2018_125_MOESM2_ESM.pdf]

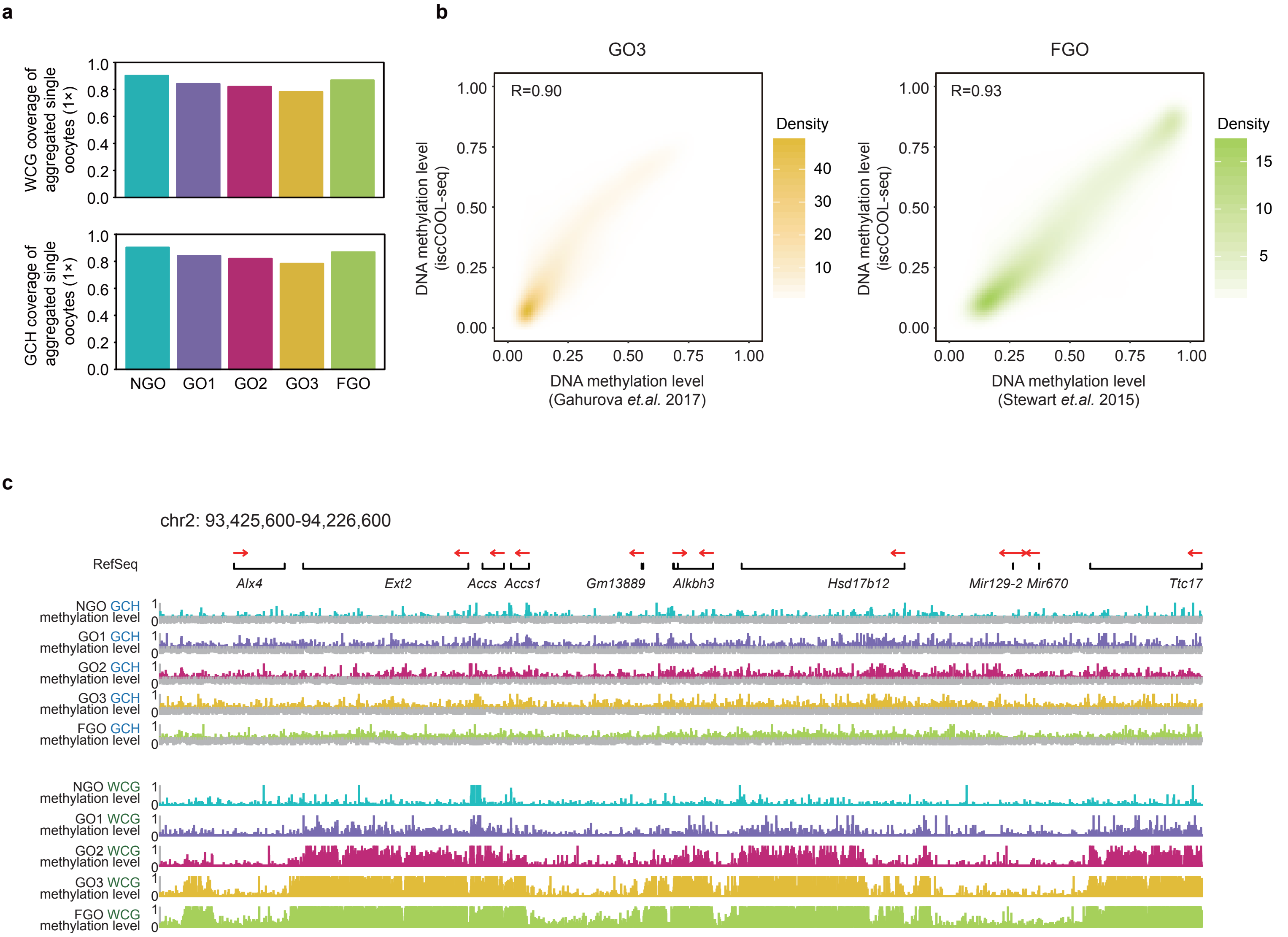

a
WCG coverage of aggregated single oocytes (1×)
GCH coverage of aggregated single oocytes (1×)
1.0
0.8
0.6
0.4
0.2
0.0
NGO
GO1
GO2
GO3
FGO
b
GO3
FGO
R=0.90
R=0.93
DNA methylation level (iscCOOL-seq)
DNA methylation level (Gahurova et.al. 2017)
DNA methylation level (Stewart et.al. 2015)
Density
0.00
0.25
0.50
0.75
1.00
10
20
30
40
5
10
15
c
chr2: 93,425,600-94,226,600
RefSeq
Alx4
Ext2
Accs
Accs1
Gm13889
Alkbh3
Hsd17b12
Mir129-2
Mir670
Ttc17
NGO GCH methylation level
GO1 GCH methylation level
GO2 GCH methylation level
GO3 GCH methylation level
FGO GCH methylation level
NGO WCG methylation level
GO1 WCG methylation level
GO2 WCG methylation level
GO3 WCG methylation level
FGO WCG methylation level

Supplementary information, Fig. S2 Data quality of single-cell multi-omic profiling of growing mouse oocytes. **(a)** The genome-wide coverage (1×) of WCGs and GCHs in aggregated single mouse oocytes by iscCOOL-seq. **(b)** Spearman's correlation of DNA methylation in the GO3 and FGO stages between the iscCOOL-seq method and the traditional PBAT method. **(c)** Representative loci showed chromatin accessibility (GCH methylation level) and DNA methylation (WCG methylation level) in growing mouse oocytes. The starting point and direction of red arrows at RefSeq genes panel represented the gene transcriptional starting site and gene transcriptional direction.
