# Supplementary material for: Integrative single-cell analysis of transcriptome, DNA methylome and chromatin accessibility in mouse oocytes
Source: Cell Res. 2018 Dec 18;29(2):110–23. doi: 10.1038/s41422-018-0125-4 (PMC6355938; doi:10.1038/s41422-018-0125-4)
Supplement: Supplementary file 3 — Supplementary information, Figure S3 [file 41422_2018_125_MOESM3_ESM.pdf]

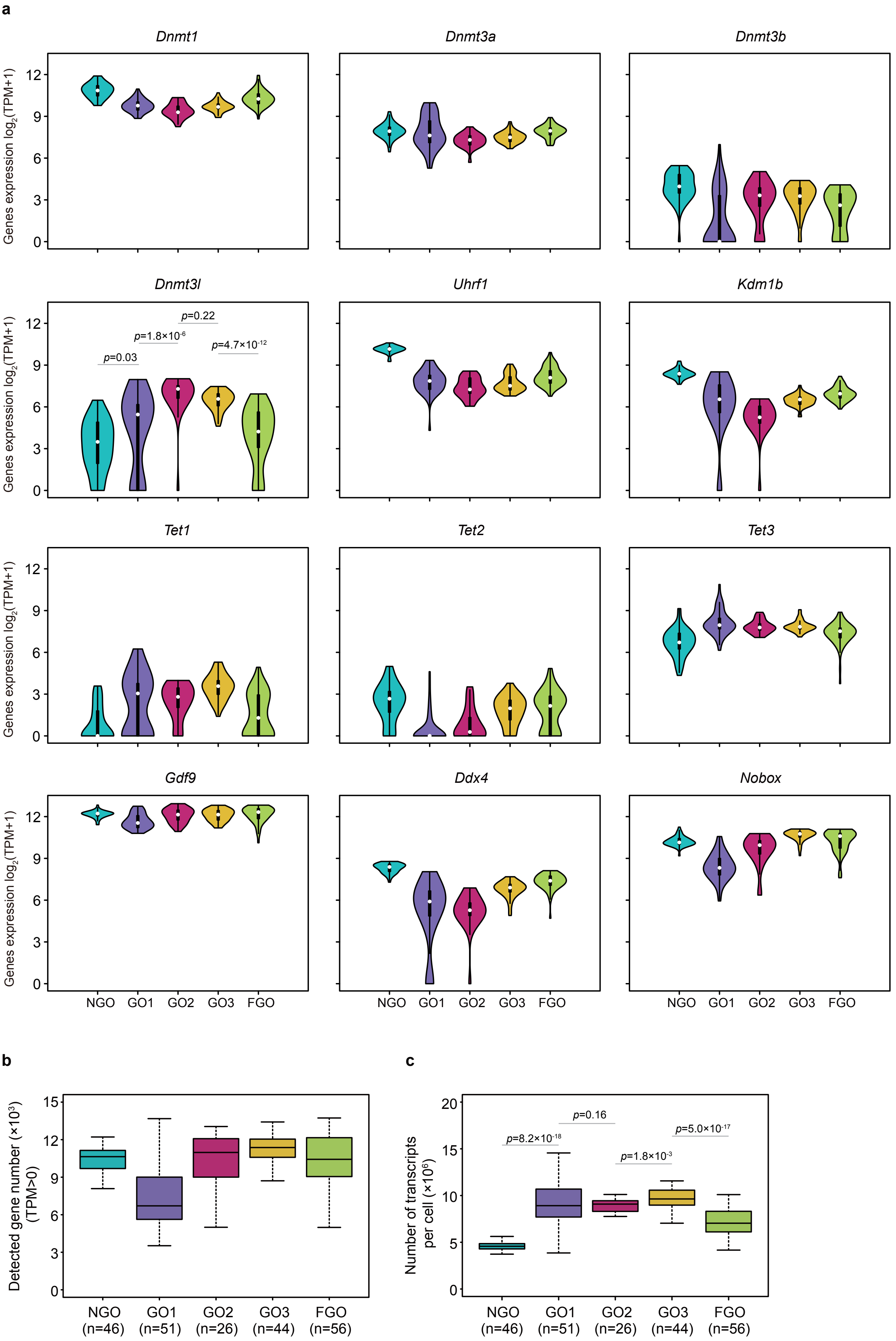
a
Dnmt1
Dnmt3a
Dnmt3b
Dnmt3l
Uhrf1
Kdm1b
Tet1
Tet2
Tet3
Gdf9
Ddx4
Nobox
Genes expression log2(TPM+1)
0
3
6
9
12
p=0.03
p=1.8×10-6
p=0.22
p=4.7×10-12
NGO
GO1
GO2
GO3
FGO
b
Detected gene number (×10³)
(TPM>0)
0
3
6
9
12
15
NGO (n=46)
GO1 (n=51)
GO2 (n=26)
GO3 (n=44)
FGO (n=56)
c
Number of transcripts
per cell (×10⁶)
0
5
10
15
20
p=8.2×10-18
p=0.16
p=1.8×10-3
p=5.0×10-17
NGO (n=46)
GO1 (n=51)
GO2 (n=26)
GO3 (n=44)
FGO (n=56)

Supplementary information, Fig. S3 Data quality of single-cell RNA-seq of growing mouse oocytes. **(a)** Representative gene expression in mouse oocytes. *Dnmt1*, *Dnmt3* and *Uhrf1* are genes related to DNA methylation. *Kdm1b* encodes a histone H3K4 demethylase required for *de novo* DNA methylation in mouse oocytes. The *Tet* family encodes a DNA dioxygenase responsible for the oxidation of 5mCs in the genome. **(b)** The number of genes detected by single-cell RNA-seq. **(c)** The relative abundance of mRNAs in single mouse oocytes calculated based on ERCC *spike-ins*. P-values were defined by the two-tailed Student's *t*-test.
