# Supplementary material for: Integrative single-cell analysis of transcriptome, DNA methylome and chromatin accessibility in mouse oocytes
Source: Cell Res. 2018 Dec 18;29(2):110–23. doi: 10.1038/s41422-018-0125-4 (PMC6355938; doi:10.1038/s41422-018-0125-4)
Supplement: Supplementary file 4 — Supplementary information, Figure S4 [file 41422_2018_125_MOESM4_ESM.pdf]

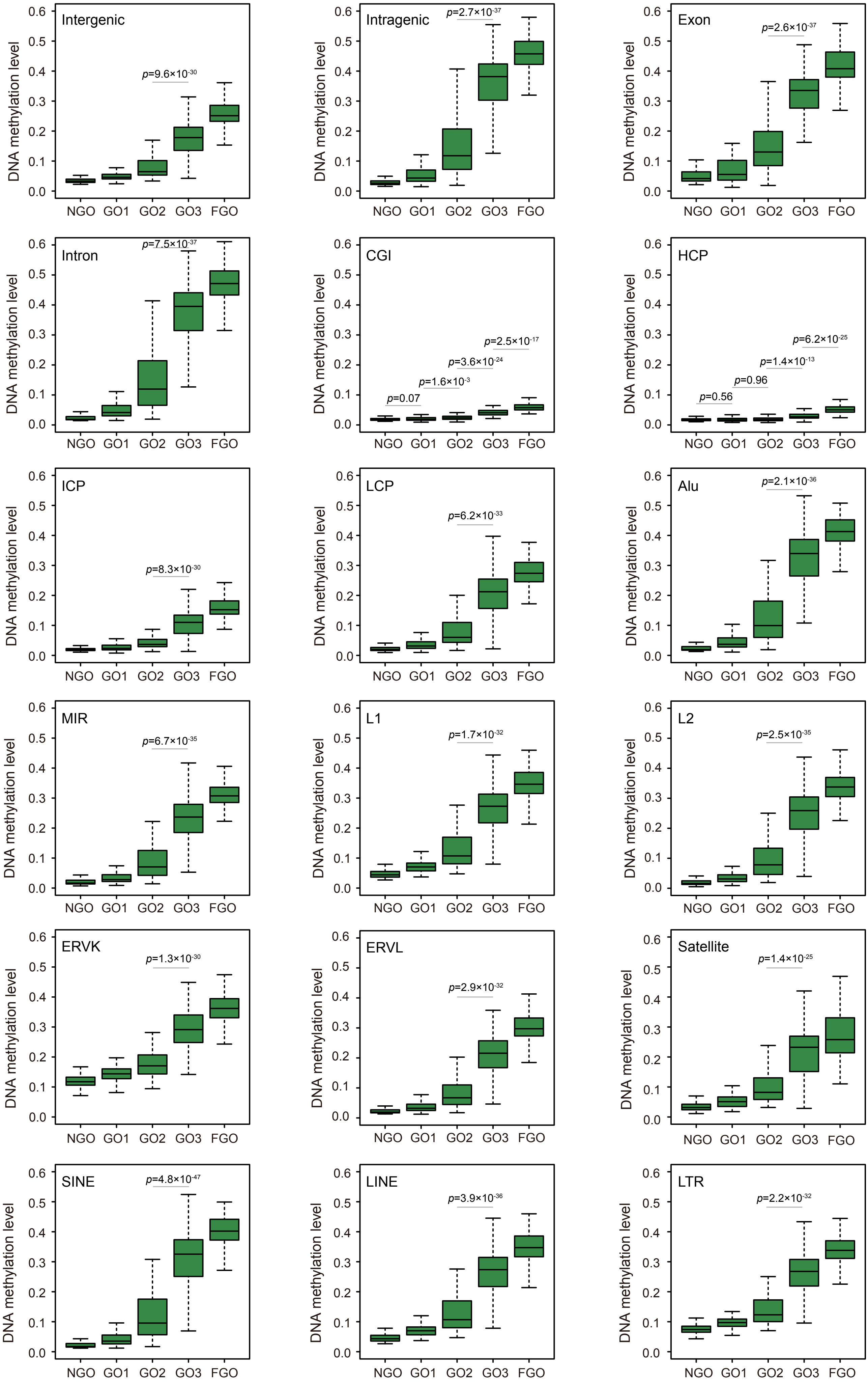
DNA methylation level
0.0 0.1 0.2 0.3 0.4 0.5 0.6
NGO GO1 GO2 GO3 FGO
Intergenic
p=9.6×10-30
Intragenic
p=2.7×10-37
Exon
p=2.6×10-37
Intron
p=7.5×10-37
CGI
p=0.07
p=1.6×10-3
p=3.6×10-24
p=2.5×10-17
HCP
p=0.56
p=0.96
p=1.4×10-13
p=6.2×10-25
ICP
p=8.3×10-30
LCP
p=6.2×10-33
Alu
p=2.1×10-36
MIR
p=6.7×10-35
L1
p=1.7×10-32
L2
p=2.5×10-35
ERVK
p=1.3×10-30
ERVL
p=2.9×10-32
Satellite
p=1.4×10-25
SINE
p=4.8×10-47
LINE
p=3.9×10-36
LTR
p=2.2×10-32

Supplementary information, Fig. S4 Dynamics of DNA methylation in single oocytes at different functional elements. Boxplot of the DNA methylation data from single oocytes was presented. P-values were defined by the two-tailed Student's *t*-test.
