# Supplementary material for: Integrative single-cell analysis of transcriptome, DNA methylome and chromatin accessibility in mouse oocytes
Source: Cell Res. 2018 Dec 18;29(2):110–23. doi: 10.1038/s41422-018-0125-4 (PMC6355938; doi:10.1038/s41422-018-0125-4)
Supplement: Supplementary file 5 — Supplementary information, Figure S5 [file 41422_2018_125_MOESM5_ESM.pdf]

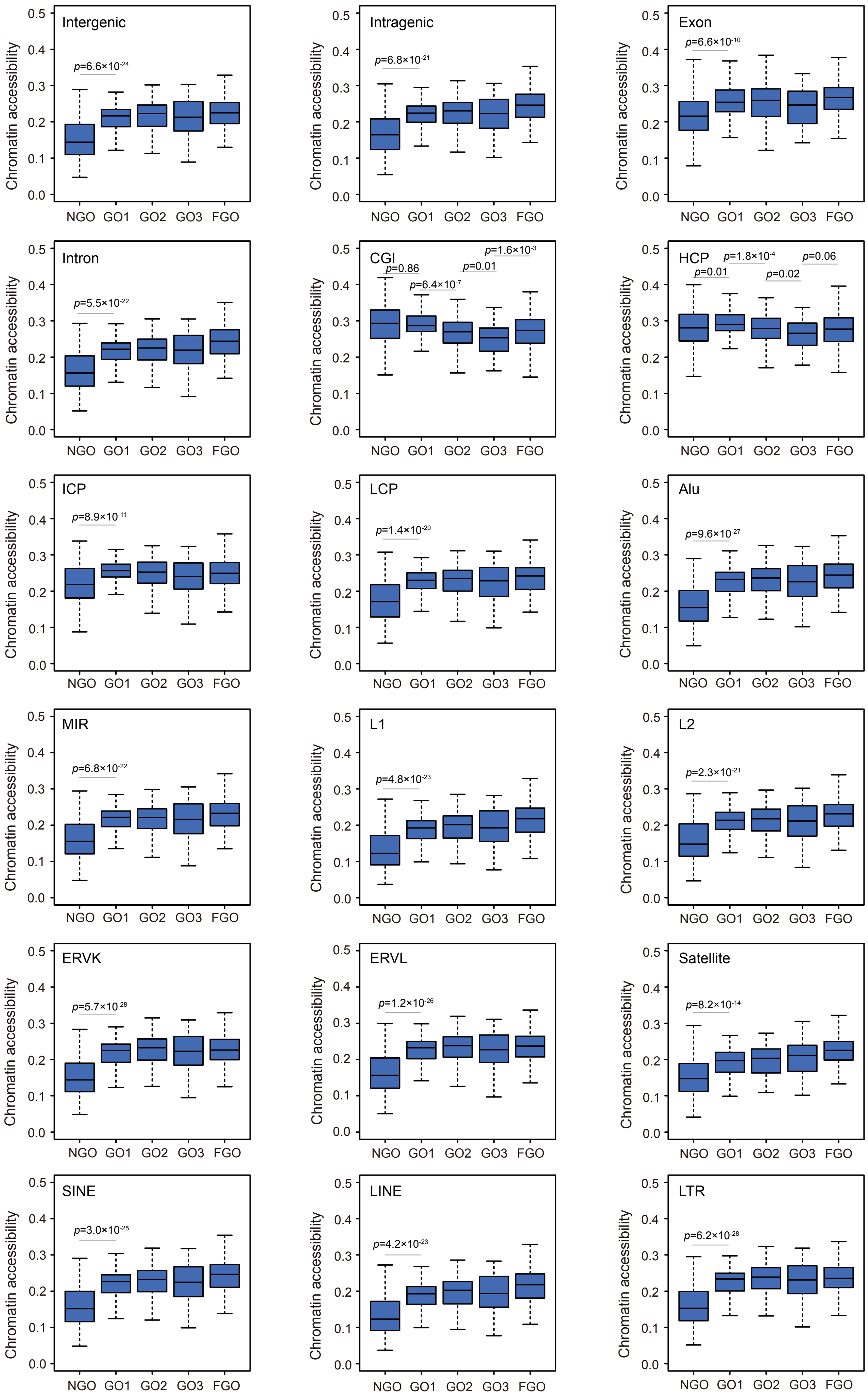

Intergenic
p=6.6×10^-24
Intragenic
p=6.8×10^-21
Exon
p=6.6×10^-10
Intron
p=5.5×10^-22
CGI
p=0.86
p=6.4×10^-7
p=0.01
p=1.6×10^-3
HCP
p=0.01
p=1.8×10^-4
p=0.02
p=0.06
ICP
p=8.9×10^-11
LCP
p=1.4×10^-20
Alu
p=9.6×10^-27
MIR
p=6.8×10^-22
L1
p=4.8×10^-23
L2
p=2.3×10^-21
ERVK
p=5.7×10^-28
ERVL
p=1.2×10^-26
Satellite
p=8.2×10^-14
SINE
p=3.0×10^-25
LINE
p=4.2×10^-23
LTR
p=6.2×10^-28
Chromatin accessibility
0.0 0.1 0.2 0.3 0.4 0.5
NGO GO1 GO2 GO3 FGO

Supplementary information, Fig. S5 Dynamics of chromatin accessibility in single oocytes at different functional elements. Boxplot of the chromatin accessibility data from single oocytes was presented. P-values were defined by the two-tailed Student's *t*-test.
