# Supplementary material for: Integrative single-cell analysis of transcriptome, DNA methylome and chromatin accessibility in mouse oocytes
Source: Cell Res. 2018 Dec 18;29(2):110–23. doi: 10.1038/s41422-018-0125-4 (PMC6355938; doi:10.1038/s41422-018-0125-4)
Supplement: Supplementary file 6 — Supplementary information, Figure S6 [file 41422_2018_125_MOESM6_ESM.pdf]

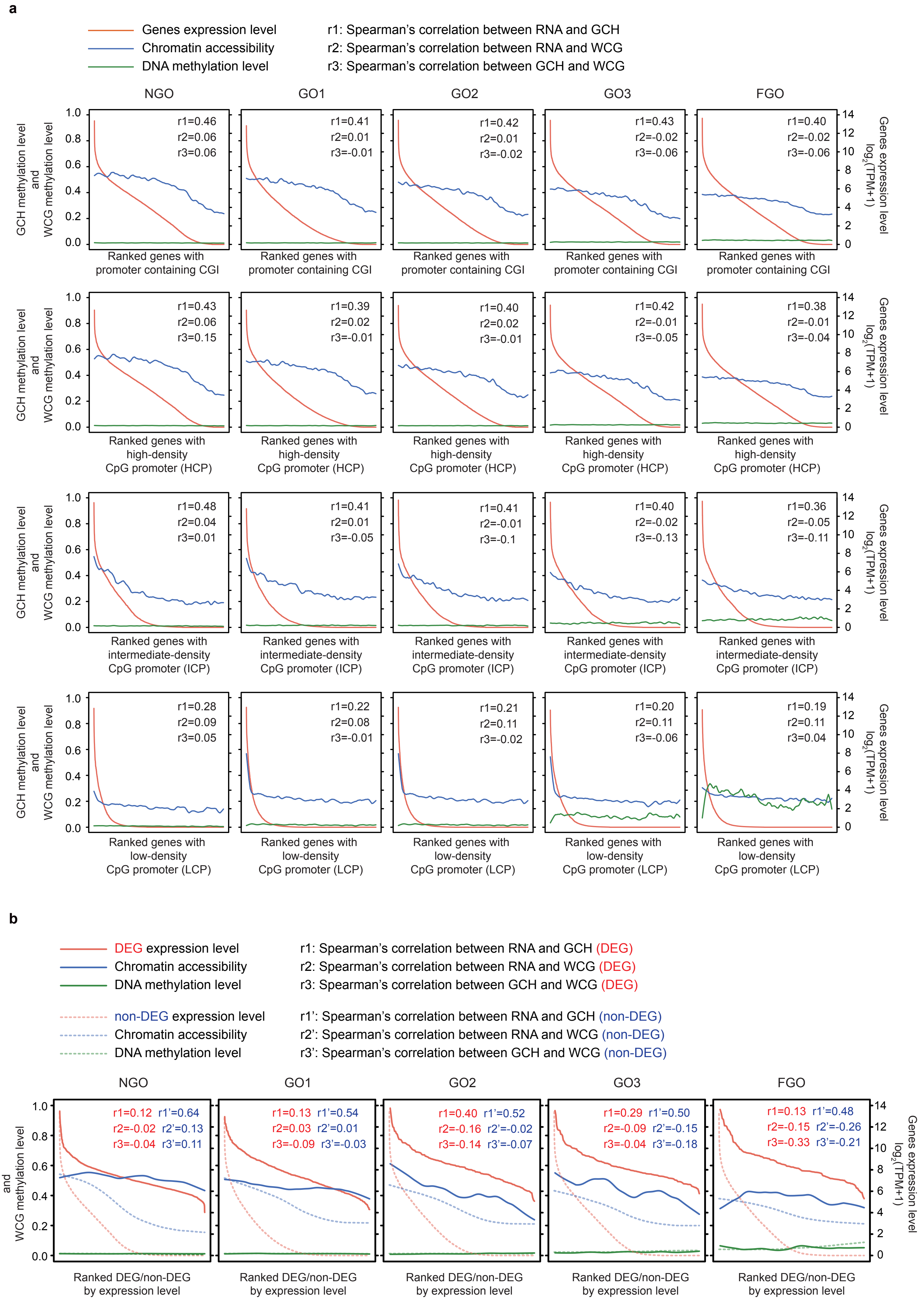
a
Genes expression level
Chromatin accessibility
DNA methylation level
r1: Spearman's correlation between RNA and GCH
r2: Spearman's correlation between RNA and WCG
r3: Spearman's correlation between GCH and WCG
NGO
GO1
GO2
GO3
FGO
GCH methylation level and WCG methylation level
Genes expression level log2(TPM+1)
r1=0.46 r2=0.06 r3=0.06
r1=0.41 r2=0.01 r3=-0.01
r1=0.42 r2=0.01 r3=-0.02
r1=0.43 r2=-0.02 r3=-0.06
r1=0.40 r2=-0.02 r3=-0.06
Ranked genes with promoter containing CGI
r1=0.43 r2=0.06 r3=0.15
r1=0.39 r2=0.02 r3=-0.01
r1=0.40 r2=0.02 r3=-0.01
r1=0.42 r2=-0.01 r3=-0.05
r1=0.38 r2=-0.01 r3=-0.04
Ranked genes with high-density CpG promoter (HCP)
r1=0.48 r2=0.04 r3=0.01
r1=0.41 r2=0.01 r3=-0.05
r1=0.41 r2=-0.01 r3=-0.1
r1=0.40 r2=-0.02 r3=-0.13
r1=0.36 r2=-0.05 r3=-0.11
Ranked genes with intermediate-density CpG promoter (ICP)
r1=0.28 r2=0.09 r3=0.05
r1=0.22 r2=0.08 r3=-0.01
r1=0.21 r2=0.11 r3=-0.02
r1=0.20 r2=0.11 r3=-0.06
r1=0.19 r2=0.11 r3=0.04
Ranked genes with low-density CpG promoter (LCP)
b
DEG expression level
Chromatin accessibility
DNA methylation level
r1: Spearman's correlation between RNA and GCH (DEG)
r2: Spearman's correlation between RNA and WCG (DEG)
r3: Spearman's correlation between GCH and WCG (DEG)
non-DEG expression level
Chromatin accessibility
DNA methylation level
r1': Spearman's correlation between RNA and GCH (non-DEG)
r2': Spearman's correlation between RNA and WCG (non-DEG)
r3': Spearman's correlation between GCH and WCG (non-DEG)
NGO
GO1
GO2
GO3
FGO
GCH methylation level and WCG methylation level
Genes expression level log2(TPM+1)
r1=0.12 r1'=0.64 r2=-0.02 r2'=0.13 r3=-0.04 r3'=0.11
r1=0.13 r1'=0.54 r2=0.03 r2'=0.01 r3=-0.09 r3'=-0.03
r1=0.40 r1'=0.52 r2=-0.16 r2'=-0.02 r3=-0.14 r3'=-0.07
r1=0.29 r1'=0.50 r2=-0.09 r2'=-0.15 r3=-0.04 r3'=-0.18
r1=0.13 r1'=0.48 r2=-0.15 r2'=-0.26 r3=-0.33 r3'=-0.21
Ranked DEG/non-DEG by expression level

Supplementary information, Fig. S6 Relationship among the chromatin accessibility and DNA methylation of promoters and the expression of the corresponding genes. **(a)** Relationship among the chromatin accessibility (200 bp upstream and 100 bp downstream of the TSS) and DNA methylation (1 kb upstream and 0.5 kb downstream of the TSS) of CGI promoters, HCP, ICP, LCP and the expression of the corresponding genes. Genes on the x-axis were ranked by the gene expression level. **(b)** Relationship among the chromatin accessibility (200 bp upstream and 100 bp downstream of the TSS) and DNA methylation (1 kb upstream and 0.5 kb downstream of the TSS) of promoters and the expression of the corresponding DEGs (differentially expressed genes), non-DEGs. Genes on the x-axis were ranked by the gene expression level.
