# Supplementary material for: Integrative single-cell analysis of transcriptome, DNA methylome and chromatin accessibility in mouse oocytes
Source: Cell Res. 2018 Dec 18;29(2):110–23. doi: 10.1038/s41422-018-0125-4 (PMC6355938; doi:10.1038/s41422-018-0125-4)
Supplement: Supplementary file 7 — Supplementary information, Figure S7 [file 41422_2018_125_MOESM7_ESM.pdf]

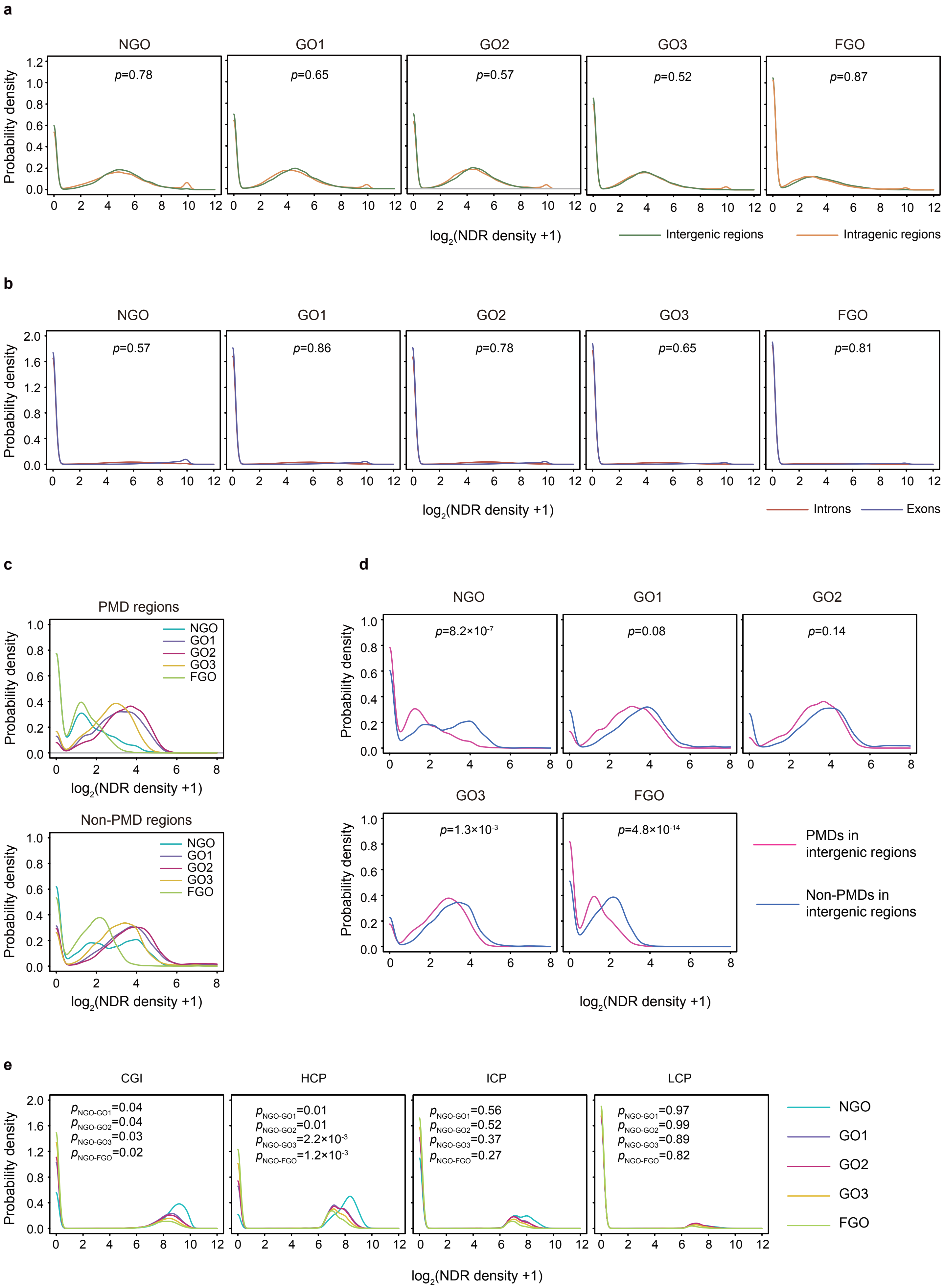
a
NGO
GO1
GO2
GO3
FGO
Probability density
p=0.78
p=0.65
p=0.57
p=0.52
p=0.87
log2(NDR density +1)
Intergenic regions
Intragenic regions
b
NGO
GO1
GO2
GO3
FGO
Probability density
p=0.57
p=0.86
p=0.78
p=0.65
p=0.81
log2(NDR density +1)
Introns
Exons
c
PMD regions
NGO
GO1
GO2
GO3
FGO
Probability density
log2(NDR density +1)
Non-PMD regions
NGO
GO1
GO2
GO3
FGO
Probability density
log2(NDR density +1)
d
NGO
GO1
GO2
Probability density
p=8.2×10-7
p=0.08
p=0.14
GO3
FGO
Probability density
p=1.3×10-3
p=4.8×10-14
PMDs in intergenic regions
Non-PMDs in intergenic regions
log2(NDR density +1)
e
CGI
HCP
ICP
LCP
Probability density
pNGO-GO1=0.04
pNGO-GO2=0.04
pNGO-GO3=0.03
pNGO-FGO=0.02
pNGO-GO1=0.01
pNGO-GO2=0.01
pNGO-GO3=2.2×10-3
pNGO-FGO=1.2×10-3
pNGO-GO1=0.56
pNGO-GO2=0.52
pNGO-GO3=0.37
pNGO-FGO=0.27
pNGO-GO1=0.97
pNGO-GO2=0.99
pNGO-GO3=0.89
pNGO-FGO=0.82
NGO
GO1
GO2
GO3
FGO
log2(NDR density +1)

Supplementary information, Fig. S7 Probability density of NDR density during oocyte growth. **(a)** Probability density plots of NDR density at intergenic and intragenic regions in growing mouse oocytes. **(b)** NDR density at introns and exons. **(c)** Probability density plots of NDR density in PMDs (upper panel) and non-PMDs (lower panel) in growing mouse oocytes. **(d)** Probability density plots of NDR density in intergenic PMDs and intergenic non-PMDs in growing mouse oocytes. **(E)** Probability density plots of NDR density at CGIs, HCPs, ICPs and LCPs. P-values were defined by the two-tailed Student's *t*-test.
