# Supplementary material for: Integrative single-cell analysis of transcriptome, DNA methylome and chromatin accessibility in mouse oocytes
Source: Cell Res. 2018 Dec 18;29(2):110–23. doi: 10.1038/s41422-018-0125-4 (PMC6355938; doi:10.1038/s41422-018-0125-4)
Supplement: Supplementary file 8 — Supplementary information, Figure S8 [file 41422_2018_125_MOESM8_ESM.pdf]

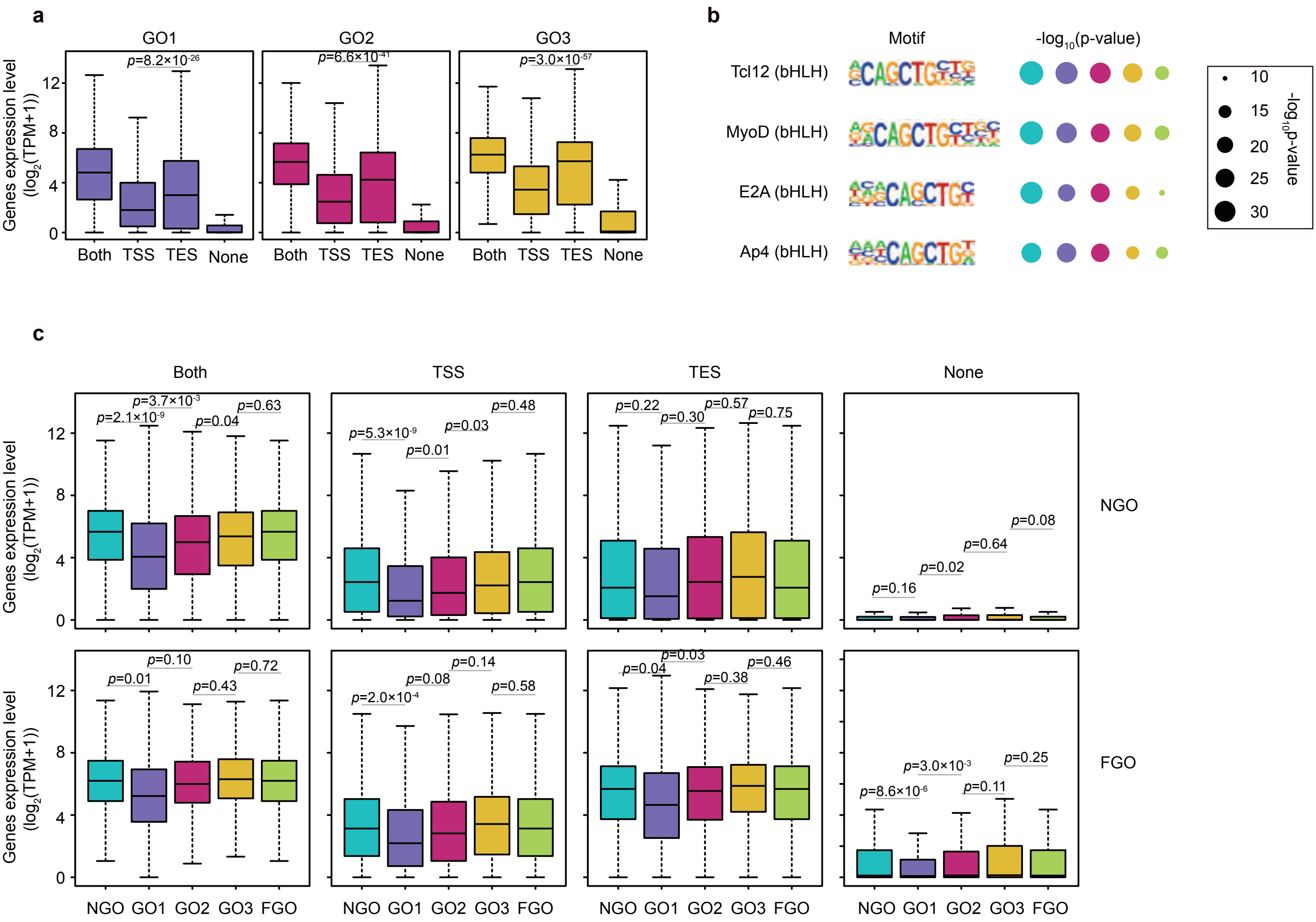
a
GO1
GO2
GO3
p=8.2×10-26
p=6.6×10-41
p=3.0×10-57
Genes expression level (log2(TPM+1))
Both TSS TES None
b
Motif
-log10(p-value)
Tcl12 (bHLH)
MyoD (bHLH)
E2A (bHLH)
Ap4 (bHLH)
10
15
20
25
30
-log10p-value
c
Both
TSS
TES
None
NGO
FGO
p=3.7×10-3
p=2.1×10-9
p=0.04
p=0.63
p=5.3×10-9
p=0.01
p=0.03
p=0.48
p=0.22
p=0.30
p=0.57
p=0.75
p=0.16
p=0.02
p=0.64
p=0.08
p=0.10
p=0.01
p=0.43
p=0.72
p=2.0×10-4
p=0.08
p=0.14
p=0.58
p=0.04
p=0.03
p=0.38
p=0.46
p=8.6×10-6
p=3.0×10-3
p=0.11
p=0.25
Genes expression level (log2(TPM+1))
NGO GO1 GO2 GO3 FGO

Supplementary information, Fig. S8 Dynamics of NDRs at TSSs and TESs during oocyte maturation. **(a)** Expression levels of genes with both TSS and TES NDRs (Both), genes with only TSS-NDRs (TSS), genes with only TES-NDRs (TES) and genes with no NDR (None) in GOs. **(b)** Analysis of motif enrichment for Tcl12, MyoD, E2A and Ap4 of the TES-NDRs from each stage. **(c)** Classification of genes into 4 groups according to the existence of a TSS-NDR or a TES-NDR in the NGO and FGO stages. The gene expression levels in single oocytes across five stages were shown. P-values were defined by the two-tailed Student's *t*-test.
