# Supplementary material for: Integrative single-cell analysis of transcriptome, DNA methylome and chromatin accessibility in mouse oocytes
Source: Cell Res. 2018 Dec 18;29(2):110–23. doi: 10.1038/s41422-018-0125-4 (PMC6355938; doi:10.1038/s41422-018-0125-4)
Supplement: Supplementary file 9 — Supplementary information, Figure S9 [file 41422_2018_125_MOESM9_ESM.pdf]

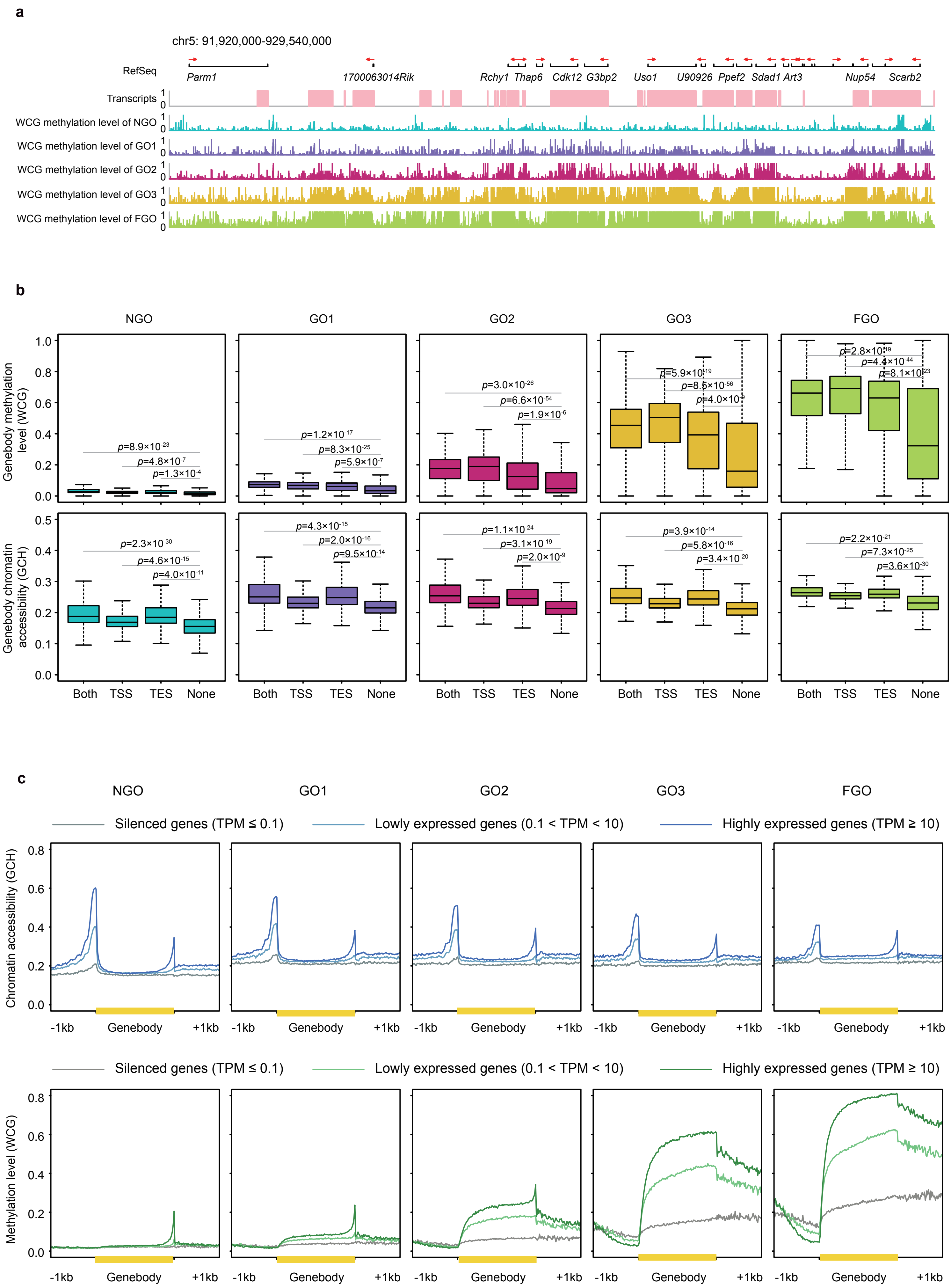
a
chr5: 91,920,000-929,540,000
RefSeq
Parm1
1700063014Rik
Rchy1
Thap6
Cdk12
G3bp2
Uso1
U90926
Ppef2
Sdad1
Art3
Nup54
Scarb2
Transcripts
WCG methylation level of NGO
WCG methylation level of GO1
WCG methylation level of GO2
WCG methylation level of GO3
WCG methylation level of FGO
b
NGO
GO1
GO2
GO3
FGO
Genebody methylation level (WCG)
p=8.9×10-23
p=4.8×10-7
p=1.3×10-4
p=1.2×10-17
p=8.3×10-25
p=5.9×10-7
p=3.0×10-26
p=6.6×10-54
p=1.9×10-6
p=5.9×10-19
p=8.5×10-56
p=4.0×10-9
p=2.8×10-19
p=4.4×10-44
p=8.1×10-23
Genebody chromatin accessibility (GCH)
p=2.3×10-30
p=4.6×10-15
p=4.0×10-11
p=4.3×10-15
p=2.0×10-16
p=9.5×10-14
p=1.1×10-24
p=3.1×10-19
p=2.0×10-9
p=3.9×10-14
p=5.8×10-16
p=3.4×10-20
p=2.2×10-21
p=7.3×10-25
p=3.6×10-30
Both
TSS
TES
None
c
NGO
GO1
GO2
GO3
FGO
Silenced genes (TPM ≤ 0.1)
Lowly expressed genes (0.1 < TPM < 10)
Highly expressed genes (TPM ≥ 10)
Chromatin accessibility (GCH)
-1kb
Genebody
+1kb
Silenced genes (TPM ≤ 0.1)
Lowly expressed genes (0.1 < TPM < 10)
Highly expressed genes (TPM ≥ 10)
Methylation level (WCG)
-1kb
Genebody
+1kb

Supplementary information, Fig. S9 Relationship among chromatin accessibility, *de novo* DNA methylation and gene expression. **(a)** Representative loci showed *de novo* DNA methylation at actively transcribed regions in oocytes. The starting point and direction of red arrows at RefSeq genes panel represented the gene transcriptional starting site and gene transcriptional direction. **(b)** DNA methylation level (upper panel) and chromatin accessibility (lower panel) of gene bodies from genes with both TSS and TES NDRs (Both), genes with only TSS-NDRs (TSS), genes with only TES-NDRs (TES) and genes with no NDR (None) in mouse oocytes. **(c)** The average chromatin accessibility (upper panel) and DNA methylation (lower panel) of RefSeq genes along gene bodies, 1 kb upstream of the TSS and 1 kb downstream of the TES at each stage. Genes were separated into three categories by their expression level. P-values were defined by the two-tailed Student's *t*-test.
