# Supplementary material for: Integrative single-cell analysis of transcriptome, DNA methylome and chromatin accessibility in mouse oocytes
Source: Cell Res. 2018 Dec 18;29(2):110–23. doi: 10.1038/s41422-018-0125-4 (PMC6355938; doi:10.1038/s41422-018-0125-4)
Supplement: Supplementary file 10 — Supplementary information, Figure S10 [file 41422_2018_125_MOESM10_ESM.pdf]

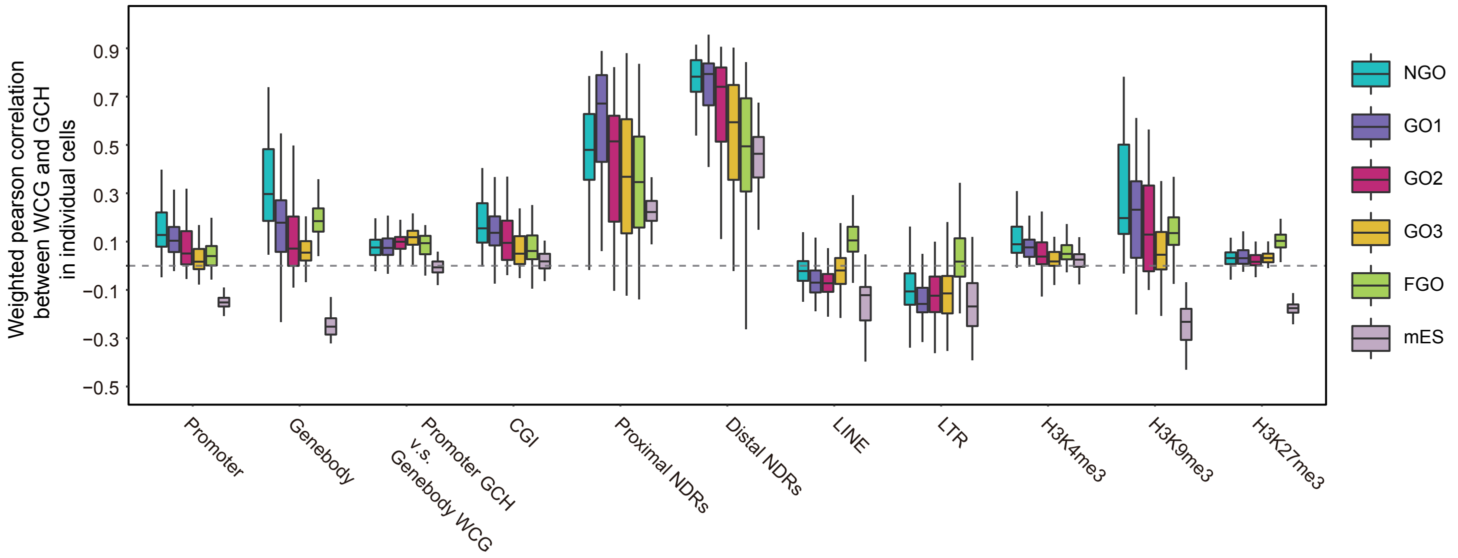
Weighted pearson correlation between WCG and GCH in individual cells
0.9
0.7
0.5
0.3
0.1
−0.1
−0.3
−0.5
Promoter
Genebody
Promoter GCH v.s. Genebody WCG
CGI
Proximal NDRs
Distal NDRs
LINE
LTR
H3K4me3
H3K9me3
H3K27me3
NGO
GO1
GO2
GO3
FGO
mES

Supplementary information, Fig. S10 Weighted Pearson correlation coefficient between WCG and GCH methylation level of individual cell at different genomic elements. The data from iscCOOL-seq of individual mouse NGOs, GOs, FGOs and ES cells were used for analysis.
