# Supplementary material for: Integrative single-cell analysis of transcriptome, DNA methylome and chromatin accessibility in mouse oocytes
Source: Cell Res. 2018 Dec 18;29(2):110–23. doi: 10.1038/s41422-018-0125-4 (PMC6355938; doi:10.1038/s41422-018-0125-4)
Supplement: Supplementary file 11 — Supplementary information, Figure S11 [file 41422_2018_125_MOESM11_ESM.pdf]

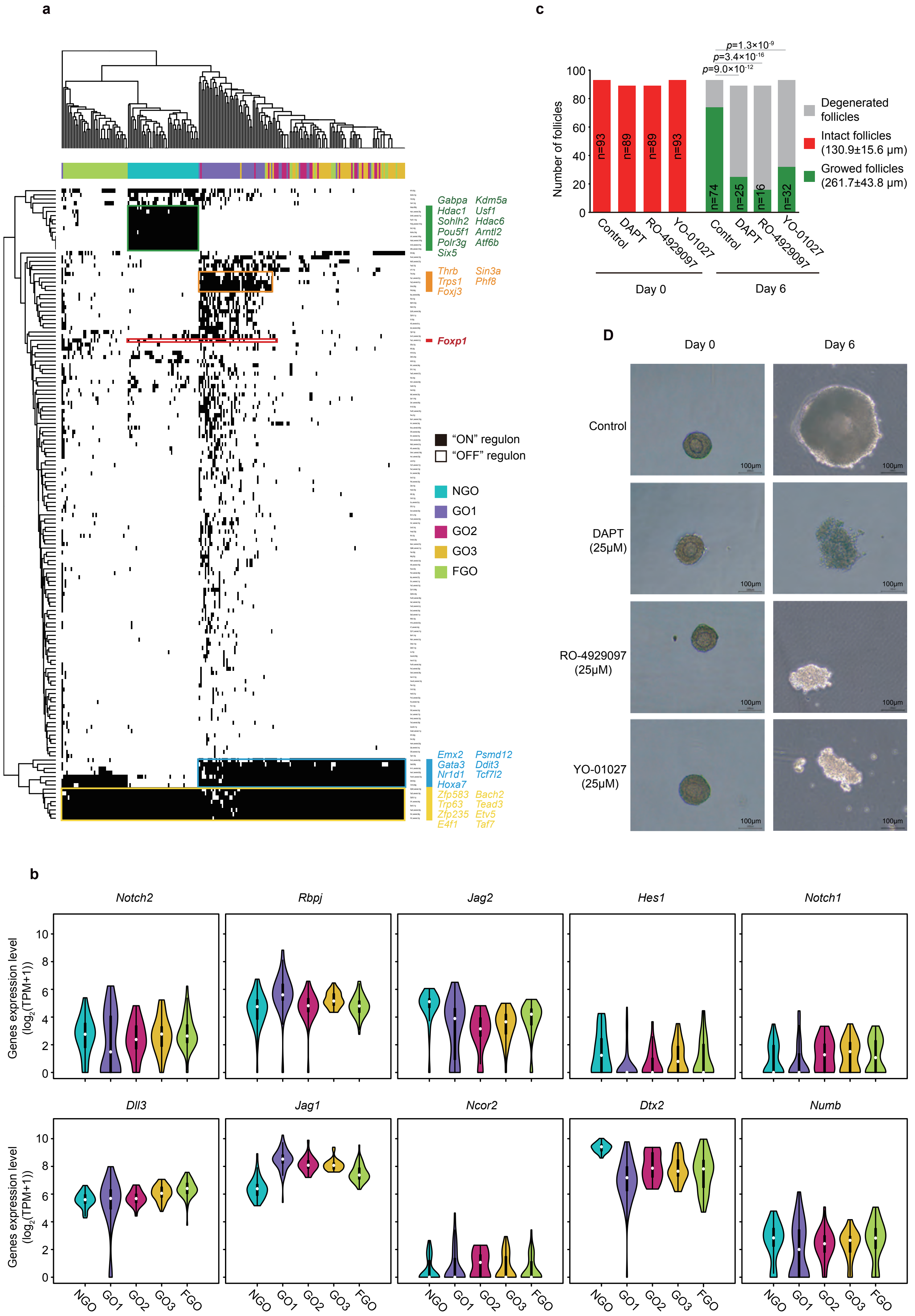
a
Gabpa Kdm5a
Hdac1 Usf1
Sohlh2 Hdac6
Pou5f1 Arntl2
Polr3g Atf6b
Six5
Thrb Sin3a
Trps1 Phf8
Foxj3
Foxp1
"ON" regulon
"OFF" regulon
NGO
GO1
GO2
GO3
FGO
Emx2 Psmd12
Gata3 Ddit3
Nr1d1 Tcf7l2
Hoxa7
Zfp583 Bach2
Trp63 Tead3
Zfp235 Etv5
E4f1 Taf7
c
p=1.3×10-9
p=3.4×10-16
p=9.0×10-12
Number of follicles
n=93
n=89
n=89
n=93
n=74
n=25
n=16
n=32
Control
DAPT
RO-4929097
YO-01027
Day 0
Day 6
Degenerated follicles
Intact follicles (130.9±15.6 μm)
Growed follicles (261.7±43.8 μm)
D
Day 0
Day 6
Control
DAPT (25μM)
RO-4929097 (25μM)
YO-01027 (25μM)
100μm
b
Notch2
Rbpj
Jag2
Hes1
Notch1
Dll3
Jag1
Ncor2
Dtx2
Numb
Genes expression level (log2(TPM+1))
NGO
GO1
GO2
GO3
FGO

Supplementary information, Fig. S11 Featured gene expression and chromatin accessibility involved in oocyte growth. **(a)** A SCENIC (Aibar *et al. Nature Methods*. 2017) heatmap showed the clustering of growing oocytes and potentially active transcription factors during oocyte maturation. scRNA-seq data was used here. **(b)** Expression levels of representative Notch signalling pathway genes in single oocytes. **(c)** Statistics of *in vitro* cultured mouse follicles either under normal condition or in the presence of notch signaling inhibitors (DAPT, RO-4929097, YO-01027). For each replicate, experiments for control and three of inhibitors groups were conducted in parallel. A total 364 of follicles from 4 replicated experiments were analysed, and the number of follicles for each group was: n = 93 for control group, n = 89 for DAPT group, n = 89 for RO-4929097 group, n = 93 for YO-01027 group. **(d)** Representative images of *in vitro* cultured mouse follicles. Scale bar indicates 100 μm. P-values were defined by the chi square test.
